# Regulation of CLC-1 chloride channel biosynthesis by FKBP8 and Hsp90β

**DOI:** 10.1038/srep32444

**Published:** 2016-09-01

**Authors:** Yi-Jheng Peng, Jing-Jia Huang, Hao-Han Wu, Hsin-Ying Hsieh, Chia-Ying Wu, Shu-Ching Chen, Tsung-Yu Chen, Chih-Yung Tang

**Affiliations:** 1 Department of Physiology, College of Medicine, National Taiwan University, Taipei, Taiwan; 2 Department of Medical Research, National Taiwan University Hospital, Taipei, Taiwan; 3 Neuroscience Center, University of California, Davis, USA; 4 Graduate Institute of Brain and Mind Sciences, College of Medicine, National Taiwan University, Taipei, Taiwan

## Abstract

Mutations in human CLC-1 chloride channel are associated with the skeletal muscle disorder myotonia congenita. The disease-causing mutant A531V manifests enhanced proteasomal degradation of CLC-1. We recently found that CLC-1 degradation is mediated by cullin 4 ubiquitin ligase complex. It is currently unclear how quality control and protein degradation systems coordinate with each other to process the biosynthesis of CLC-1. Herein we aim to ascertain the molecular nature of the protein quality control system for CLC-1. We identified three CLC-1-interacting proteins that are well-known heat shock protein 90 (Hsp90)-associated co-chaperones: FK506-binding protein 8 (FKBP8), activator of Hsp90 ATPase homolog 1 (Aha1), and Hsp70/Hsp90 organizing protein (HOP). These co-chaperones promote both the protein level and the functional expression of CLC-1 wild-type and A531V mutant. CLC-1 biosynthesis is also facilitated by the molecular chaperones Hsc70 and Hsp90β. The protein stability of CLC-1 is notably increased by FKBP8 and the Hsp90β inhibitor 17-allylamino-17-demethoxygeldanamycin (17-AAG) that substantially suppresses cullin 4 expression. We further confirmed that cullin 4 may interact with Hsp90β and FKBP8. Our data are consistent with the idea that FKBP8 and Hsp90β play an essential role in the late phase of CLC-1 quality control by dynamically coordinating protein folding and degradation.

CLC-1 chloride (Cl^−^) channels are essential for setting the membrane excitability of skeletal muscle, where the Cl^−^ channels are estimated to contribute up to 70–80% of the resting membrane conductance^1^,^2^,^3^. More than 100 different mutations in the *CLCN1* gene, which encodes the human voltage-gated CLC-1 Cl^−^ channel, have been associated with the hereditary muscle disorder myotonia congenita that is characterized by muscle stiffness after voluntary contraction^4^,^5^,^6^,^7^. One of the myotonia congenita-associated mutations involves a conservative alanine-to-valine mutation (A531V) located at the transmembrane helix O of the human CLC-1 channel^8^. The A531V mutation is found in significant prevalence in northern Finland and northern Scandinavia^8^,^9^. Unlike many myotonia-related *CLCN1* mutations that result in notably altered gating functions of CLC-1 channels^10^,^11^,^12^,^13^, we previously demonstrated that the gating property of the A531V mutant is similar to that of its wild-type (WT) counterpart^14^. Nonetheless, the mutant channel displays a dramatically diminished whole-cell current density and a considerable reduction in the total protein level, both of which can be attributed to enhanced protein degradation^14^,^15^. Reduced protein expression and defective membrane trafficking may also be associated with other myotonia-related CLC-1 mutations^16^,^17^.

The A531V mutant appears to be endowed with a folding anomaly that makes the mutant channel undesirable for the protein quality control system in endoplasmic reticulum (ER), thereby tilting the biosynthetic balance toward the degradation pathway. It is still unclear, however, how the ER quality control system and the ER-associated degradation (ERAD) system recognize and process disease-associated mutant CLC-1 proteins. We recently reported that two cullin (CUL)-really interesting new gene (RING) E3 ubiquitin ligases, CUL4A/B-damage-specific DNA binding protein 1 (DDB1)-cereblon (CRBN) E3 ligase complexes, catalyze the polyubiquitination and promote the degradation of CLC-1 channels^18^. Therefore, one further step to addressing the molecular pathophysiology of myotonia congenita is to elucidate the interplay between the protein quality control system and the CUL4A/B-DDB1-CRBN complex-mediated degradation pathway.

A cardinal process during protein biogenesis involves conformation surveillance of nascent polypeptide by a network of molecular chaperones and cofactors (co-chaperones) that efficiently assist protein folding, thereby minimizing degradation/aggregation of proteins in nonnative states^19^,^20^,^21^. In this study, we aim to investigate the molecular nature of the chaperone/co-chaperone network monitoring the biosynthesis of human CLC-1. We demonstrate that CLC-1 channels are associated with the molecular chaperones heat shock cognate protein 70 (Hsc70) and heat shock protein 90β (Hsp90β), as well as their co-chaperones FK506-binding protein 8 (FKBP8; also known as FKBP38), activator of Hsp90 ATPase homolog 1 (Aha1), and Hsp70/Hsp90 organizing protein (HOP). Biochemical and electrophysiological characterizations reveal that these co-chaperones and chaperones enhance both the protein level and the functional expression of CLC-1 WT and A531V mutant. Importantly, we present additional evidence suggesting that, in addition to promoting CLC-1 protein folding, FKBP8 and Hsp90β may also play a critical role in regulating CLC-1 degradation by the CUL4A/B-DDB1-CRBN complex.

## Results

### Promotion of CLC-1 protein level by co-chaperones

**Figure 1:**
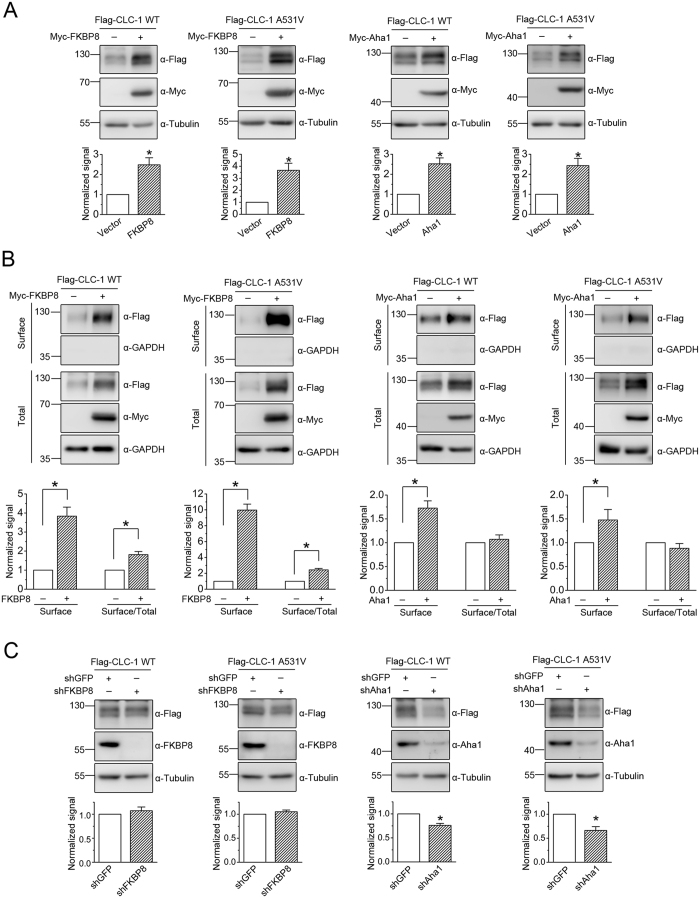
Effects of FKBP8 and Aha1 on protein expression and membrane trafficking of CLC-1. (**A**) (*Top*) Representative immunoblots showing the effect of Myc-tagged FKBP8 and Aha1 on protein level of Flag-tagged CLC-1 channels. Co-expression with the Myc vector was used as the vector control. Expressions of tubulin are displayed as the loading control. Proteins were detected with the indicated antibodies (α-Myc, α-Flag, or α-Tubulin). The molecular weight markers (in kiloDaltons) are labeled to the left. (*Bottom*) Quantification of relative CLC-1 level. Protein density was standardized as the ratio of CLC-1 signal to cognate tubulin signal. Values from the FKBP8/Aha1 co-expression group (*hatched bars*) were then normalized to those for the corresponding vector control (*clear bars*). (**B**) Surface biotinylation experiments on HEK293T cells expressing Flag-CLC-1. (*Top*) Representative immunoblots. Cell lysates from biotinylated intact cells were either directly employed for immunoblotting analyses (total) or subject to streptavidin pull-down prior to immunoblotting analyses (surface). Expressions of GAPDH are displayed as the loading control. (*Bottom*) Quantification of surface protein level (Surface) and membrane trafficking efficiency (Surface/Total). The surface protein density was standardized as the ratio of surface signal to cognate total GAPDH signal, followed by normalization to that of the corresponding vector control. The total protein density was standardized as the ratio of input signal to GAPDH signal. The membrane trafficking efficiency was expressed as surface protein density divided by the corresponding standardized total protein density. The mean ratios in the presence of FKBP8/Aha1 were normalized to those of corresponding vector controls. (**C**) shRNA knock-down of endogenous FKBP8/Aha1 in HEK293T cells. The shRNA for GFP was used as the lentiviral infection control. Expressions of tubulin are displayed as the loading control. The protein density was standardized as the ratio of Flag-CLC-1 signal to cognate total tubulin signal, followed by normalization to that of the corresponding GFP control. Asterisks denote significant difference from the control (*, *t*-test: p < 0.05). See Supplementary Table S1 for more details on quantification values. The gels were run under the same experimental conditions. Uncropped images of immunoblots are shown in Supplementary Fig. S4.

To study the molecular nature of CLC-1 protein quality control system, we began by searching for potential CLC-1-binding partners that are previously demonstrated to play a role in the chaperone/co-chaperone network. By performing yeast two-hybrid screening of a mouse skeletal muscle cDNA library with a bait sequence corresponding to an intracellular carboxyl-terminal region of the human CLC-1 channel (see Supplementary Methods), we identified the co-chaperones FKBP8 and Aha1. FKBP8 is an Hsp90-associated membrane-anchored immunophilin with potential peptidyl-prolyl *cis-trans* isomerase function, whereas Aha1 is a cytosolic protein regulating the ATPase activity of Hsp90^19^,^22^,^23^,^24^. Furthermore, both FKBP8 and Aha1 have been demonstrated to play critical roles in ER quality control of cystic fibrosis transmembrane conductance regulator (CFTR) Cl^−^ channels^25^,^26^,^27^,^28^. The interaction of CLC-1 with FKBP8/Aha1 was further confirmed by GST pull-down assay (see Supplementary Methods) with GST fusion proteins comprising C-terminal regions of CLC-1 (GST-CLC-1-C1, -C2, and -C3) (Suppl. Fig. 1A), and by immunoprecipitation experiment with full-length CLC-1 channel (Suppl. Fig. 1B). Over-expression of FKBP8/Aha1 substantially increases the protein level of CLC-1 WT and A531V mutant heterologously expressed in HEK293T cells (Fig. 1A) (Suppl. Table S1). CLC-1 surface expression, as determined by the surface biotinylation assay, is also significantly promoted by both FKBP8 and Aha1 (Fig. 1B) (Suppl. Table S1). Moreover, the membrane trafficking efficiency of CLC-1, which was quantified by the ratio of surface expression to total protein level, appears to be enhanced by FKBP8, but not by Aha1 (Fig. 1B) (Suppl. Table S1). Interestingly, FKBP8, but not Aha1, seems to display a more prominent effect on the A531V mutant than on its WT counterpart. For CLC-1 WT, FKBP8 co-expression leads to about 2.5-fold and 3.8-fold increase in the total protein and surface expression, respectively. For the A531V mutant, however, FKBP8 co-expression results in about 3.7-fold and 9.9-fold increase in the total protein and surface expression, respectively. On the other hand, upon down-regulating endogenous FKBP8/Aha1 level in HEK293T cells with the RNA interference technique, we found that CLC-1 protein level is effectively reduced by lentiviral infection with the shRNA for Aha1, but not by that for FKBP8 (Fig. 1C) (Suppl. Table S1).

**Figure 2:**
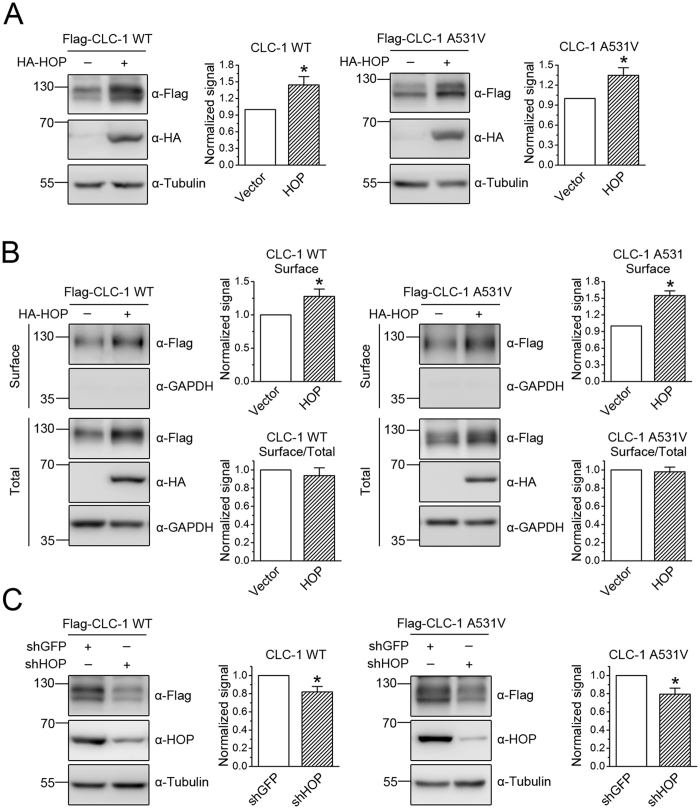
Effects of HOP on protein expression and membrane trafficking of CLC-1. (**A**) Representative immunoblots showing the effect of HA-tagged HOP on CLC-1 protein level. The mean relative protein levels for WT (n = 10) and A531V (n = 13) are about 1.4 and 1.3, respectively. (**B**) HOP co-expression on surface protein level and membrane trafficking efficiency of CLC-1. The mean relative surface expression ratios for WT (n = 5) and A531V (n = 3) are about 1.3 and 1.5, respectively. The mean relative membrane trafficking ratios for WT and A531V are about 0.9 and 1.0, respectively. (**C**) shRNA knock-down of endogenous HOP in HEK293T cells. The mean relative protein levels for WT (n = 17) and A531V (n = 11) are about 0.8 and 0.8, respectively. Asterisks denote significant difference from the control (*, *t*-test: p < 0.05). The gels were run under the same experimental conditions. Uncropped images of immunoblots are shown in Supplementary Fig. S4.

CLC-2 is a ubiquitously expressed Cl^−^ channel that, along with CLC-1, belongs to the CLC Cl^−^ channel family^29^. In a different set of experiments in which we performed yeast two-hybrid screening of a rat brain cDNA library with a carboxyl-terminal region of mouse CLC-2, we identified the co-chaperone HOP. HOP is a soluble protein mediating the interaction of Hsp70 and Hsp90, as well as regulating Hsp90 ATPase activity^19^,^22^. In addition, HOP has been implicated in ER-associated folding of CFTR as well^25^. To test the idea that HOP may also contribute to CLC-1 protein quality control, we first employed GST pull-down and immunoprecipitation assays to verify the interaction of CLC-1 with HOP (Suppl. Fig. 2A). Heterologous expression studies in HEK293T cells further confirm that HOP promotes both the total protein level and the surface expression of CLC-1 WT and A531V mutant (Fig. 2A,B). Similar to the result observed for Aha1, HOP fails to discernibly affect the membrane trafficking of CLC-1 (Fig. 2B). Furthermore, also like Aha1, shRNA knock-down of endogenous HOP in HEK293T cells leads to a notable reduction of CLC-1 protein level (Fig. 2C).

Together, these observations suggest that the co-chaperones FKBP8, Aha1, and HOP promote CLC-1 biosynthesis. In addition, our data seem to imply that the role of FKBP8 in CLC-1 protein quality control may be considerably different from that of Aha1 and HOP.

### Facilitation of CLC-1 biosynthesis by chaperones

**Figure 3:**
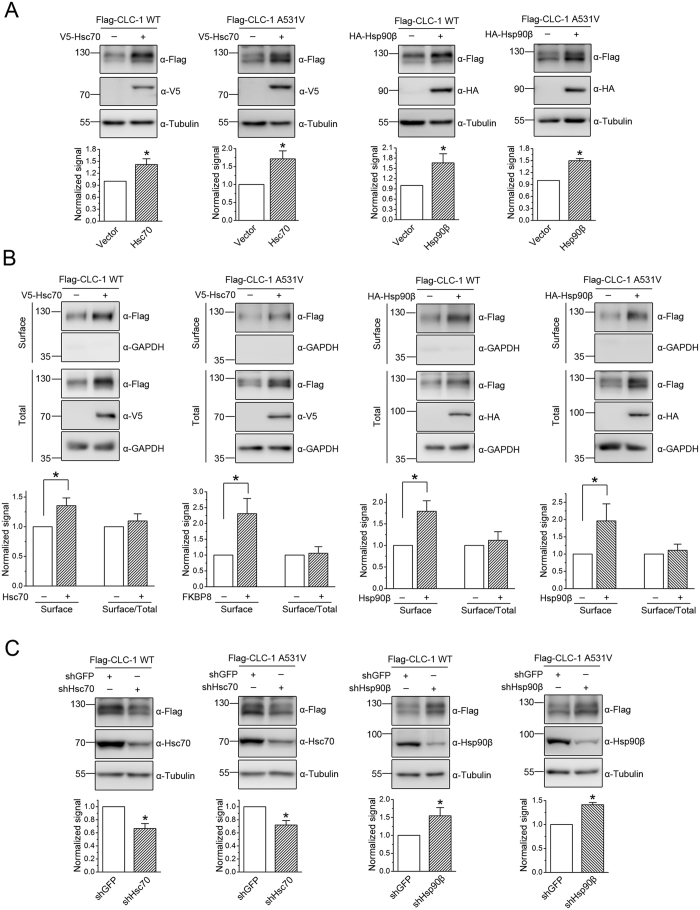
Effects of Hsc70 and Hsp90β on protein expression and membrane trafficking of CLC-1. **(A)** Representative immunoblots showing the effect of V5-tagged Hsc70 or HA-tagged Hsp90β co-expression on CLC-1 protein level. The mean relative protein levels for WT (n = 9–10) are about 1.4 (Hsc70) and 1.6 (Hsp90β); those for A531V (n = 11–13) are about 1.7 (Hsc70) and 1.5 (Hsp90β). **(B)** Hsc70 or Hsp90β co-expression on surface protein level and membrane trafficking efficiency of CLC-1. The mean relative surface expression ratios for WT (n = 4) are about 1.4 (Hsc70) and 1.8 (Hsp90β); those for A531V (n = 4) are about 2.3 (Hsc70) and 2.4 (Hsp90β). The mean relative membrane trafficking ratios for WT are about 1.1 (Hsc70) and 1.2 (Hsp90β); those for A531V are about 1.1 (Hsc70) and 1.3 (Hsp90β). **(C)** shRNA knock-down of endogenous Hsc70 or Hsp90β in HEK293T cells. The mean relative protein levels for WT (n = 6–15) are about 0.7 (Hsc70) and 1.5 (Hsp90β); those for A531V (n = 6–13) are about 0.7 (Hsc70) and 1.4 (Hsp90β). Asterisks denote significant difference from the control (*, *t*-test: p < 0.05). The gels were run under the same experimental conditions. Uncropped images of immunoblots are shown in Supplementary Fig. S4.

FKBP8, Aha1, and HOP serve as co-chaperones for the interconnected Hsp70 and Hsp90 molecular chaperone systems^19^,^22^,^30^. Moreover, a recent report employing mass spectrometry combined with luminescence-based mammalian interactome assays indicates that the co-chaperone FKBP8 may interact more strongly with Hsp90β (the constitutive Hsp90 isoform) than with Hsp90α (the inducible Hsp90 isoform)^31^. We therefore went on to investigate the potential role of the constitutively expressed chaperone isoforms Hsc70 and Hsp90β in CLC-1 biogenesis. Representative GST pull-down and immunoprecipitation data illustrated in Supplementary Fig. 2B indicate that, in HEK293T cells, CLC-1 may be physically associated or at least stably co-exist in the same protein complex with Hsc70. Over-expression of Hsc70 significantly increases both the protein level and the surface expression of CLC-1 WT and A531V mutant, but does not appreciably alter the membrane trafficking efficiency of the Cl^−^ channel (Fig. 3A,B). Conversely, suppressing endogenous Hsc70 expression in HEK293T cells with shRNA prominently reduces CLC-1 protein level (Fig. 3C).

The interaction between CLC-1 and Hsp90β was only demonstrated by GST pull-down (Suppl. Fig. 2C), but not by immunoprecipitation, suggesting that the two protein molecules probably form transient association with each other. Similar to the effect of Hsc70, Hsp90β promotes CLC-1 protein level and surface expression, but not membrane trafficking (Fig. 3A,B), suggesting that both Hsc70 and Hsp90β facilitate CLC-1 biosynthesis. Since over-expressing the inducible chaperone isoforms Hsp70/Hsp90α fails to discernibly increase CLC-1 protein level (data not shown), the foregoing observations do not appear to result from non-specific CLC-1 responses to Hsc70/Hsp90β over-expression in HEK293T cells. Surprisingly, shRNA knock-down of endogenous Hsp90β in HEK293T cells results in a remarkable enrichment of the protein level of CLC-1 WT and A531V mutant (Fig. 3C), which may be caused by a unknown compensatory response to Hsp90β suppression in HEK293T cells. Alternatively, this result may imply a differential role between Hsc70 and Hsp90β in CLC-1 quality control system.

### Regulation of CLC-1 degradation by FKBP8 and Hsp90β

**Figure 4:**
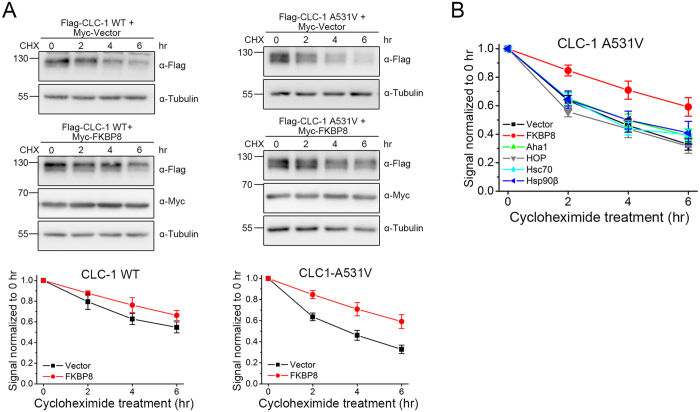
FKBP8 improves CLC-1 protein stability. Characterization of CLC-1 protein turn-over kinetics in HEK293T cells by employing different treatment durations of 100 μg/ml cycloheximide (CHX). **(A)** (*Top*) Representative immunoblots showing the effect of FKBP8 co-expression. Co-expression with the Myc vector was used as the control experiment. (*Bottom*) Quantification of CLC-1 protein half-life. Protein densities were normalized to those of corresponding no-treatment controls at 0 hr. **(B)** Quantification of the effect of co-chaperone/chaperone co-expression on A531V protein half-life. See Supplementary Table S2 for more details on the estimated protein half-life values. The gels were run under the same experimental conditions. Uncropped images of immunoblots are shown in Supplementary Fig. S4.

Given the regulatory role of ER quality control system in protein homeostasis (proteostasis), an increase in the net expression level can be attributed to an enhanced chaperone-assisted folding of native protein and/or a reduced chaperone-directed degradation of misfolded protein^19^,^20^,^21^,^32^. To address whether the abovementioned co-chaperones and chaperones may control protein degradation of CLC-1, next we examined their role in CLC-1 protein stability by performing the cycloheximide chase experiment. Figure 4A and Supplementary Table S2 exemplify the effect of FKBP8 over-expression on the protein degradation time course of CLC-1 WT and A531V mutant in HEK293T cells. FKBP8 effectively raises the protein half-life of CLC-1 WT from about 6.6 to 10.1 hours. Furthermore, for the A531V mutant that is associated with enhanced proteasomal degradation^14^, FKBP8 considerably increases its protein half-life from about 3.6 to 8.0 hours. We then investigated the effect of over-expressing Aha1, HOP, Hsc70, or Hsp90β. Figure 4B and Supplementary Table S2 show that only FKBP8 is capable of significantly improving protein stability of the A531V mutant, suggesting that FKBP8 is remarkably effective in correcting the protein folding defect of the CLC-1 mutant.

**Figure 5:**
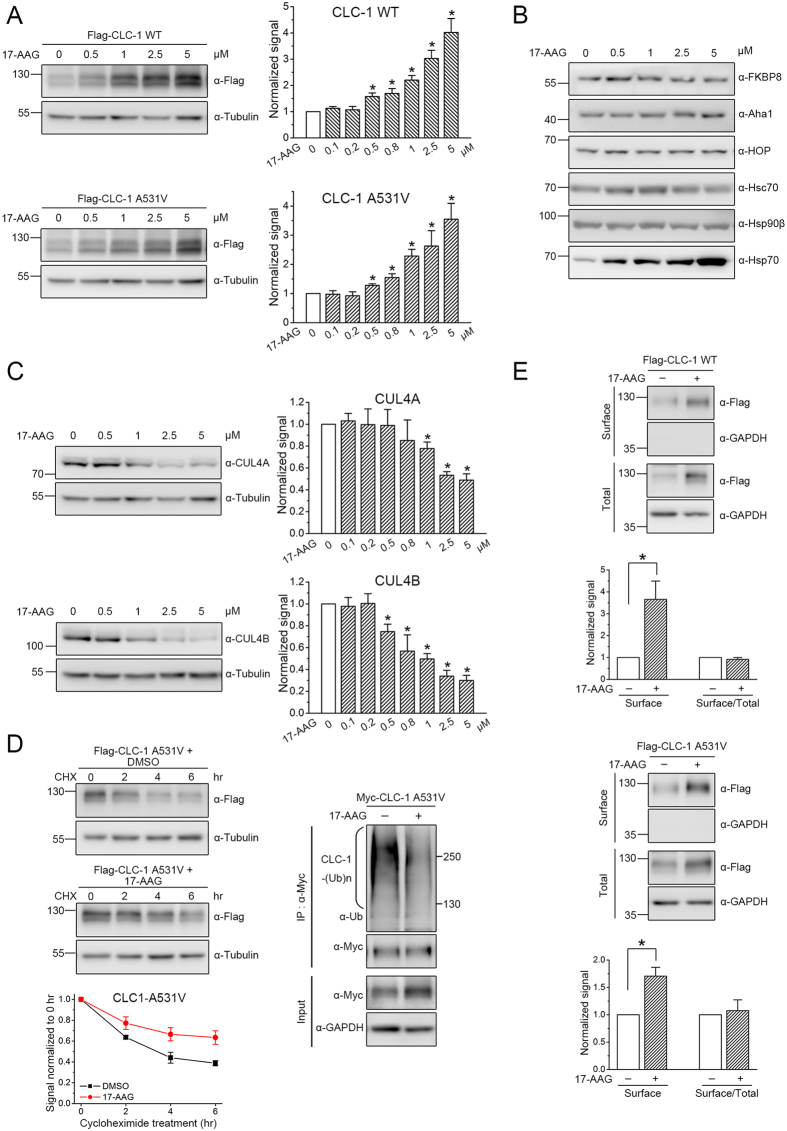
17-AAG reduces CLC-1 protein degradation. Biochemical demonstration of the regulation of CLC-1 WT and A531V mutant by 17-AAG treatment in HEK293T cells. **(A)** (*Left*) Representative immunoblots showing the effect of 17-AAG on CLC-1 protein level. DMSO treatment (0 μM) was used as the control experiment. (*Right*) Quantification of relative CLC-1 protein levels in response to different concentrations of 17-AAG. **(B**) The effect of 17-AAG on endogenous FKBP8, Aha1, HOP, Hsc70, Hsp90β, and HSP70 levels in HEK293T cells. **(C)** The effect of 17-AAG on endogenous CUL4A and CUL4B levels in HEK293T cells. See Supplementary Table S3 for more details on the quantification of 17-AAG effects on protein levels. **(D)** (*Left*) The effect of 1 μM 17-AAG on protein turn-over kinetics of Flag-CLC-1 A531V mutant. DMSO treatment was used as the control experiment. See Supplementary Table S2 for more details on the estimated protein half-life values. (*Right*) Representative immunoblots showing the effect of 1 μM 17-AAG on protein ubiquitination of Myc-CLC-1 A531V mutant. Cell lysates were immunoprecipitated with the anti-Myc antibody, and CLC-1 polyubiquitination [CLC-1-(Ub)n] by endogenous ubiquitin was identified by immunoblotting the immunoprecipitates with the anti-ubiquitin (Ub) antibody. **(E)** The effect of 1 μM 17-AAG on surface protein level (Surface) and membrane trafficking efficiency (Surface/Total) of CLC-1. The mean ratios were normalized to those of the corresponding DMSO controls. The mean relative surface expression ratios (n = 4) are about 3.6 (WT) and 1.7 (A531V). The mean relative membrane trafficking ratios are about 0.9 (WT) and 1.1 (A531V). Asterisks denote significant difference from the control (*, *t*-test: p < 0.05). The gels were run under the same experimental conditions. Uncropped images of immunoblots are shown in Supplementary Fig. S4.

In addition to its common role in stabilizing client proteins, Hsp90β may also promote the degradation of misfolded client proteins by directly interacting with E3 ubiquitin ligases^33^,^34^,^35^. In other words, depending on the mechanistic role of Hsp90β in the ER quality control system of its client proteins, pharmacological inhibition of Hsp90β function may lead to either enhanced or reduced protein degradation^36^,^37^,^38^. To gain further insight into the role of Hsp90β in the proteostatic mechanism of CLC-1, we assessed the effect of the Hsp90 inhibitor 17-allylamino-17-demethoxygeldanamycin (17-AAG), which suppresses the ATPase activity by blocking ATP binding to Hsp90^39^,^40^. Figure 5A and Supplementary Table S3 demonstrate that, in HEK293T cells, treatment with 17-AAG for 24 hours dramatically increases the protein level of both CLC-1 WT and A531V mutant in a concentration-dependent manner. A simple interpretation of this result appears to be that, rather than assisting the formation of mature CLC-1 conformation, Hsp90β promotes the degradation of the Cl^−^ channel. Nevertheless, given that Hsp90β may interact with multiple client proteins in HEK293T cells, an alternative possibility is that the observed CLC-1 up-regulation arises from 17-AAG-induced disruption of Hsp90β interaction with endogenous client protein(s) in HEK293T cells, which in turn alters CLC-1 proteostasis. To test the latter hypothesis, we examined the effect of 17-AAG treatment on endogenous FKBP8, Aha1, HOP, Hsc70, and Hsp90β levels in HEK293T cells. Figure 5B and Supplementary Table S3 clearly show that 17-AAG fails to noticeably affect the protein expression of these co-chaperones and chaperones. Pharmacological inhibition of Hsp90 with geldanamycin or 17-AAG is known to induce prominent up-regulation of Hsp70 (Fig. 5B) (Suppl. Table S3)^41^,^42^; however, as mentioned above, over-expressing Hsp70 did not appreciably increase CLC-1 protein level, suggesting that, at least in HEK293T cells, up-regulation of Hsp70 does not seem to contribute to the enhancement of CLC-1 expression by 17-AAG. Since suppression of endogenous CUL4A/B with either dominant-negative CUL4 mutants or shRNA for CUL4 effectively enhances CLC-1 expression in HEK293T cells^18^, we then examined the effect of 17-AAG treatment on endogenous CUL4A/B expression in HEK293T cells. Figure 5C and Supplementary Table S3 depict that 17-AAG indeed significantly decreases endogenous CUL4A/B expression in HEK293T cells, with CUL4B showing a more prominent concentration-dependent reduction pattern. Moreover, similar to the effect of FKBP8 over-expression, 17-AAG treatment markedly raises the protein half-life of the A531V mutant from about 3.8 to 8.4 hours, which can be attributed to reduced polyubiquitination of CLC-1 channels (Fig. 5D) (Suppl. Table S2). However, unlike FKBP8, 17-AAG treatment fails to discernibly alter the membrane trafficking efficiency of CLC-1 (Fig. 5E).

**Figure 6:**
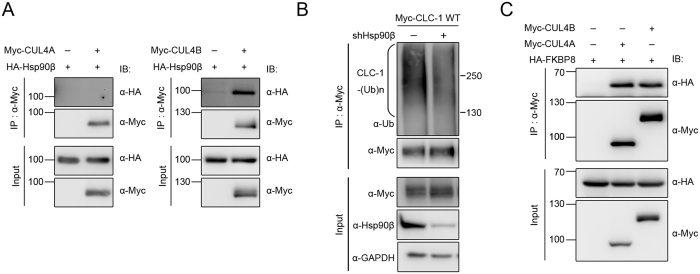
Cullin 4 co-exists in the same protein complex with Hsp90β and FKBP8. Interaction of cullin 4 with Hsp90β and FKBP8 in HEK293T cells. **(A)** Co-immunoprecipitation of Myc-CUL4B, but not Myc-CUL4A, with HA-Hsp90β. **(B)** shRNA knock-down of Hsp90β reduces CLC-1 polyubiquitination. **(C)** Co-immunoprecipitation of Myc-CUL4A and Myc-CUL4B with HA-FKBP8. Corresponding expression levels of CUL4A/B, Hsp90β, and FKBP8 in the lysates are shown in the *Input* lane. Input represents about 10% of the total protein used for immunoprecipitation. The gels were run under the same experimental conditions. Uncropped images of immunoblots are shown in Supplementary Fig. S4.

The foregoing data strongly suggest that Hsp90 may be essential for stabilizing CUL4A/B. A previous study involving luminescence-based interactome assays suggests that Hsp90 may directly interact with CUL4B^43^. To confirm this potential interaction between CUL4 and Hsp90β in HEK293T cells, we went on to perform immunoprecipitation experiments. Figure 6A shows that CUL4B, but not CUL4A, can be effectively co-immunoprecipitated with Hsp90β in HEK293T cells. Importantly, shRNA knock-down of endogenous Hsp90β in HEK293T cells significantly enhances CLC-1 protein level (see Fig. 3C). Consistent with this notion, shRNA knock-down of endogenous Hsp90β in HEK293T cells results in a noticeable interruption of the polyubiquitination of CLC-1 (Fig. 6B). Together, these observations imply that suppression of Hsp90β function may vigorously down-regulate CUL4 expression in HEK293T cells, consequently reducing CLC-1 degradation. Furthermore, results from immunoprecipitation experiments support the idea that FKBP8 may also co-exist in the same protein complex with CUL4A/B (Fig. 6C). Therefore, when we consider the overall impact of shRNA knock-down or pharmacological inhibition of Hsp90β/FKBP8 on CLC-1 proteostasis, the effect of disrupted CLC-1 folding may be virtually diminished or even out-balanced by that of reduced CLC-1 degradation.

### Enhancement of CLC-1 current level by co-chaperones and chaperones

**Figure 7:**
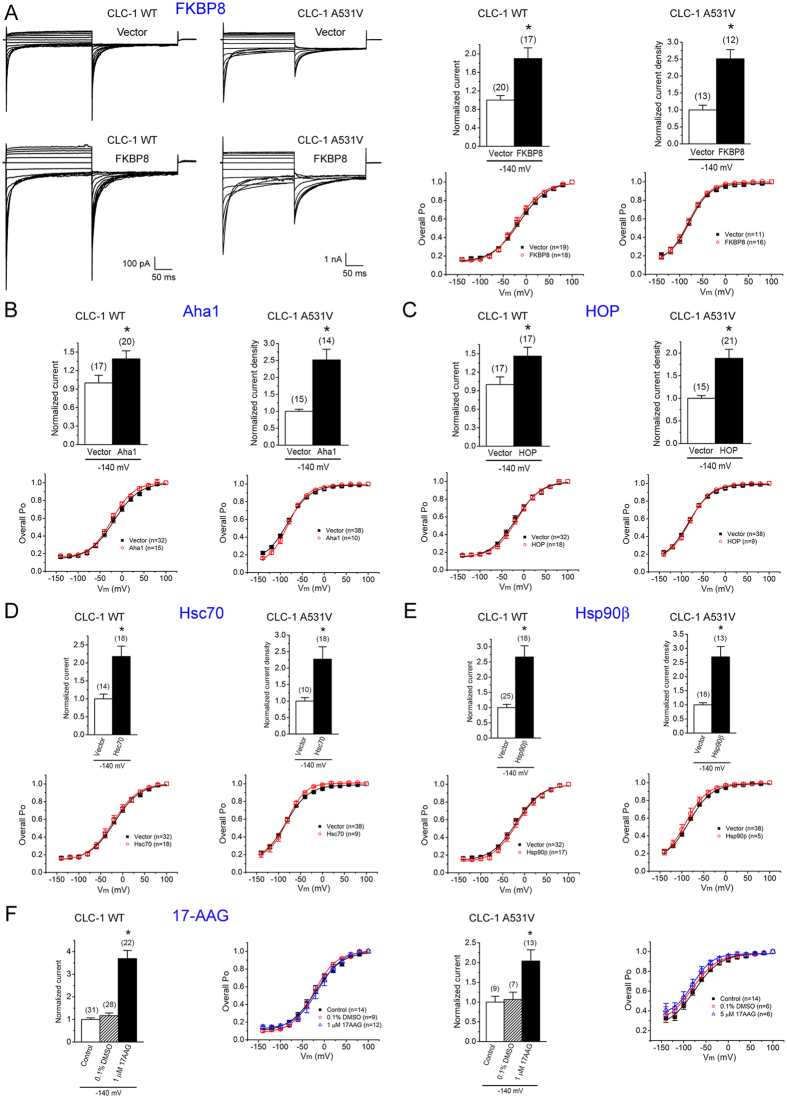
Co-chaperones and chaperones increase the functional expression of CLC-1. Electrophysiological analyses of Flag-CLC-1 WT and A531V mutant channels in HEK293T cells. **(A)** The effect of FKBP8 co-expression. (*Left panels*) Representative cell-attached and whole-cell patch clamp recordings of WT and A531V, respectively. The holding potential was 0 mV. The voltage protocol comprised a 200-ms test pulse (Vm) ranging from +100 mV to −140 mV in −20 mV steps, followed by a second voltage step (tail potential) to −100 mV for 200 ms. (*Upper right panels*) Instantaneous cell-attached current amplitudes (WT) or whole-cell current densities (A531V) at the test pulse potential of −140 mV were used for normalization with respect to the corresponding vector control. The mean relative current levels for WT and A531V are about 1.9 and 2.5, respectively. (*Lower right panels*) Steady-state voltage-dependence of the open probability (*P*_o_–V curve) of CLC-1 channels. Compared to CLC-1 WT, the A531V mutant displays an apparently left-shifted *P*_o_–V curve, which primarily arises from the different cytoplasmic ATP concentrations between cell-attached (WT) and whole-cell (A531V) configurations (refer to Suppl. Fig. S3 for experimental evidence). See Supplementary Methods for more details on the analysis of *P*_o_–V curves. **(B**) The effect of Aha1 co-expression. The mean relative current levels for WT and A531V are about 1.4 and 2.5, respectively. **(C)** The effect of HOP co-expression. The mean relative current levels for WT and A531V are about 1.5 and 1.9, respectively. **(D)** The effect of Hsc70 co-expression. The mean relative current levels for WT and A531V are about 2.2 and 2.3, respectively. **(E)** The effect of Hsp90β co-expression. The mean relative current levels for WT and A531V are about 2.7 and 2.7, respectively. **(F)** The effect of treatment with 1 μM 17-AAG. The mean relative current levels for WT and A531V are about 3.5 and 2.0, respectively. Two types of drug-free incubation (control and 0.1% DMSO) were used to verify the effect of 17-AAG. Asterisks denote a significant difference from the control condition (*, *t*-test: p < 0.05).

One critical question that remains unanswered is whether the increased protein biosynthesis induced by the abovementioned co-chaperones and chaperones really corresponds to enhanced amount of surface CLC-1 protein capable of forming functional Cl^−^ channels. To address this question, we studied the effect of the co-chaperones and chaperones on CLC-1 functional expression in HEK293T cells. Due to their drastically different protein level, CLC-1 WT and A531V mutant were subject to cell-attached and whole-cell patch clamp analyses, respectively^14^,^18^. Figure 7A illustrates that FKBP8 over-expression results in remarkable augmentation of cell-attached current amplitude and whole-cell current density of CLC-1 WT and A531V mutant, respectively. Consistent with the aforementioned biochemical observations (Fig. 1) (Suppl. Table S1), the functional enhancement effect of FKBP8 is slightly more prominent for the A531V mutant (~2.5-fold increase) than for CLC-1 WT (~1.9-fold increase). Moreover, FKBP8 over-expression does not appreciably alter the steady-state voltage-dependence (*P*_o_–V curve) of CLC-1 currents, suggesting that FKBP8 probably induces up-regulation of CLC-1 channels endowed with mature and correct protein conformation. Similar current enhancement effects were also observed when we co-expressed CLC-1 with Aha1, HOP, Hsc70, or Hsp90β (Fig. 7B–E). Furthermore, treatment with the Hsp90 inhibitor 17-AAG also effectively increases the functional expression of CLC-1 WT and A531V mutant (Fig. 7F).

## Discussion

Anomalous protein maturation arising from disrupted ER quality control, excessive ERAD, or defective membrane trafficking has been implicated in the molecular pathogenesis of many ion channel diseases (channelopathies)^21^,^44^. Given that the myotonia congenita-related A531V mutant is associated with enhanced protein degradation and defective membrane trafficking properties^14^,^15^, it is imperative to decipher the molecular machinery essential for protein biosynthesis of CLC-1. The discovery of the CUL4A/B-DDB1-CRBN complex as the E3 ubiquitin ligase catalyzing CLC-1 polyubiquitination^18^ prompted us to further identify the conformation surveillance mechanism regulating CLC-1 proteostasis. In this study, we show that the co-chaperones FKBP8, Aha1, and HOP, as well as the constitutively expressed chaperones Hsc70 and Hsp90β, may directly interact with CLC-1 Cl^−^ channels. Over-expression of these co-chaperones and chaperones substantially promotes the biosynthesis and functional expression of CLC-1 WT and A531V mutant. Conversely, shRNA knock-down of endogenous Aha1, HOP, or Hsc70 expression in HEK293T cells results in notable down-regulation of CLC-1 protein level, consistent with idea that Aha1, HOP, and Hsc70 are responsible for facilitating CLC-1 protein folding in the quality control system.

By contrast, shRNA knock-down of endogenous FKBP8 level in HEK293T cells does not appreciably change CLC-1 protein level. Moreover, infection with shRNA for Hsp90β prominently enhances CLC-1 biosynthesis. These apparently paradoxical results appear to imply that FKBP8 and Hsp90β may play additional roles in the CLC-1 quality control system. Consistent with this notion, suppression of Hsp90β function with 17-AAG dramatically down-regulates endogenous CUL4 expression in HEK293T cells, thereby reducing polyubiquitination and degradation of CLC-1. In addition, like Hsp90β, FKBP8 co-exists in the same protein complex with CUL4, raising the possibility that FKBP8 may also contribute to the stabilization of CUL4 by Hsp90β. Importantly, FKBP8 displays three unique features: 1) a more prominent biosynthesis-enhancement effect on the A531V mutant than on its WT counterpart (see Fig. 1A, Suppl. Table S1, and Fig. 7A); 2) effective improvement of CLC-1 protein stability (see Fig. 4 and Suppl. Table S2), and 3) significant promotion of CLC-1 membrane trafficking efficiency (see Fig. 1B and Suppl. Table S1). Therefore, our results appear to suggest that FKBP8 plays a decisive role in correcting protein folding defect of CLC-1.

**Figure 8:**
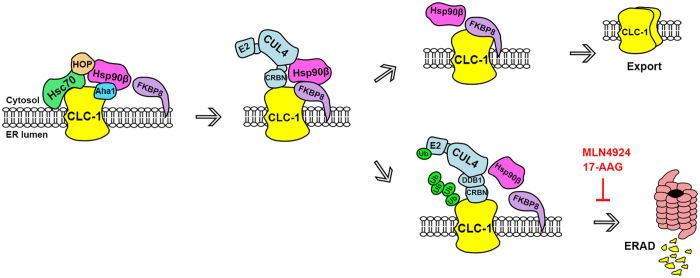
A model of the ER quality control system for CLC-1 channel. In this schematic diagram of CLC-1 biogenesis process, the ER protein quality control system is hypothesized to comprise the co-chaperones FKBP8, Aha1, and HOP, as well as the constitutively expressed chaperones Hsc70 and Hsp90β. Hsc70 and HOP may assist the early stage of CLC-1 folding, whereas Aha1, Hsp90β, and FKBP8 may promote the late stage of CLC-1 folding. FKBP8 is further proposed to be essential for determining whether CLC-1 protein can be properly folded for subunit assembly and thereafter exported for membrane trafficking. In addition, Hsp90β and FKBP8 may regulate ER-associated degradation (ERAD) of CLC-1 by interacting with the cullin 4 E3 ligase complex that catalyzes the covalent linkage of ubiquitin (Ub) to CLC-1. The degradation of CLC-1 can be effectively attenuated by the cullin E3 ligase blocker MLN4924, as well as the Hsp90 inhibitor 17-AAG.

Taken together, we suggest a model of CLC-1 proteostasis mechanism that is schematically represented in Fig. 8. The inferred protein folding pathway for CLC-1 resembles the ER quality control model previously proposed for another Cl^−^ channel, CFTR^25^,^27^,^28^,^45^,^46^. We propose that Hsc70 and HOP may assist the early stage of CLC-1 folding before passing the channel protein to the Hsp90β cycle, wherein Aha1, Hsp90β, and FKBP8 work in concert to further promote CLC-1 folding. We hypothesize that FKBP8 is responsible for the last stage of protein folding and is essential for determining whether CLC-1 protein can be properly exported for membrane trafficking. The major difference between our scheme and the CFTR model concerns the coupling between molecular chaperones and the protein degradation pathway. One of the best characterized E3 ubiquitin ligases for CFTR is carboxyl terminus of Hsc70-interacting protein (CHIP); misfolded CFTR is thought to be recognized through Hsc70-CHIP interaction and thereafter subject to CHIP-mediated polyubiquitination^45^,^47^. By contrast, the present report suggests that Hsp90β and FKBP8 may play an additional role in regulating CLC-1 degradation by interacting with CUL4. We therefore suggest that misfolded CLC-1 may be primarily processed by Hsp90β/FKBP8-CUL4 interaction. Nevertheless, we cannot rule out the possibility that Hsc70 may also be essential for CLC-1 ubiquitination mediated by as yet unknown E3 ligases.

The majority of our conclusions are based on biochemical experiments using heterologous expression system. One potential limitation with immunoblotting analyses involving protein over-expression is that the sensitivity of the protein detection system from time to time needs to be reduced to avoid signal saturation. Consequently, it is likely that we may occasionally overlook the contribution of chaperones/co-chaperones imparting minute effects on CLC-1 biosynthesis. This potential bias against weaker protein signals may additionally contribute to the quantitative discrepancy between immunoblotting and electrophysiological analyses reported in this study. Due to the voltage clamp capacity of the signal amplifier for the patch clamp system, there is a stringent limitation on the size of the CLC-1 current amplitudes that can be properly recorded; in other words, cells with higher CLC-1 protein expression levels are more likely to be excluded from our functional data analyses. Nevertheless, the results derived from immunoblotting and electrophysiological analyses do agree nicely on a qualitative basis. Another related issue on electrophysiology concerns the fact that CLC-1 WT currents were recorded using the cell-attached configuration. As we reported previously, under the whole-cell mode, CLC-1 currents from WT channels were recorded 4–7 hours post-transfection; by contrast, no significant Cl^−^ currents were observed for A531V until 8–11 hours post-transfection^14^. The current amplitudes of A531V reach a steady-state level at about 24 hours post-transfection, at which whole-cell WT current amplitudes invariably exceed the range of optimal voltage clamp efficiency for the patch amplifier system. This problem can only be overcome by a dramatic reduction of the amount of CLC-1 cDNA used for transfection, which, however, would render it virtually impossible to effectively co-express any chaperone or co-chaperone protein. On the other hand, a likely caveat associated with the cell-attached configuration is that CLC-1 proteins may not be evenly distributed over the cell surface. To minimize the impact of this potential problem, we incorporated a large number of WT membrane patches (ranging from 10 to 31; see Fig. 7) for statistical analyses. Moreover, the validity of the WT data collected with the cell-attached configuration was confirmed by repeating the identical co-expression experiments with the A531V mutant using the whole-cell mode.

One of the surprising findings of our research is the promotion of CLC-1 biosynthesis by the Hsp90 inhibitor 17-AAG. The suppressive effect of 17-AAG treatment on CUL4A/B protein level does not seem to be a special case for HEK293T cells, as a similar result was also observed in HeLa cells treated with a chemical analogue of 17-AAG^37^. Together these data are consistent with the idea that, in human cells, Hsp90β is essential for the stabilization of CUL4. Further investigations are required to elucidate the detailed mechanisms underlying how 17-AAG treatment leads to down-regulation of CUL4. Since Hsp90β is a binding partner of CUL4B, but not CUL4A (see Fig. 6A)^43^, we speculate that Hsp90β may indirectly stabilize CUL4A through other client protein(s). Interestingly, FKBP8 has been shown to interact with the S2 subunit of the 19S proteasome in HEK293T cells and may contribute to anchoring a subset of the proteasome complexes to the organellar membrane of mitochondria and ER in various cell lines^48^. Therefore, it remains to be determined whether FKBP8 may facilitate protein folding/stability of CUL4 via certain Hsp90-dependent and/or -independent processes. Overall, our findings seem to raise an intriguing possibility that Hsp90β may serve as a molecular hub that facilitates the association of CLC-1 channels with Aha1, FKBP8, and CUL4, thereby dynamically coupling the protein folding and degradation pathways of CLC-1 biosynthesis.

Another fascinating discovery arising from the present study is that only FKBP8 can pronouncedly enhance the membrane trafficking efficiency of CLC-1 channels. Emerging evidence indicates that membrane-bound protein is also susceptible to stringent conformation surveillance and substantial degradation by the endosomal-lysosomal pathway, a mechanism known as peripheral quality control^49^,^50^. FKBP8 is usually considered as an internal membrane protein resident at mitochondria and ER^23^,^24^. In line with this notion, FKBP8 was previously suggested to contribute to the ER biogenesis, but not the peripheral quality control, of CFTR^51^. However, an alternative interpretation of the unique membrane trafficking effect of FKBP8 on CLC-1 is that the co-chaperone may additionally contribute to the peripheral quality control of CLC-1 localized at the plasma membrane. Future experiments will be required to address whether FKBP8, as well as the other CLC-1-related co-chaperones, chaperones, or even E3 ubiquitin ligase complex proteins, may also be involved in the peripheral quality control of CLC-1 WT and A531V mutant. In addition, as defective biosynthesis appears to contribute to other myotonia congenita mutations^16^,^17^, it is critical to investigate whether FKBP8 may also improve the protein expression and membrane trafficking of the other disease-related CLC-1 mutant channels.

Recent advances in the molecular elucidation of defective protein biosynthesis mechanisms underlying numerous human diseases drive the developments of novel therapeutic strategies aiming at adapting proteostasis networks to restore normal physiology^32^,^52^. For example, a substantial amount of different chemical compounds have been developed to correct the biosynthetic anomaly of disease-associated mutant CFTR proteins^53^,^54^. Current treatment for myotonia primarily focuses on the symptomatic relief of enhanced muscle tone with action potential-reducing agents such as Mexiletine, which exerts use-dependent block of surface voltage-gated Na^+^ channels (Na_V1.4_) in skeletal muscles^55^,^56^,^57^. No CLC-1-targeting drug is available at present. Previously, we provided the first evidence showing that CLC-1 ubiquitination is suppressed by MLN4924, which blocks the neddylation of cullin E3 ligases and has emerged as an anti-cancer agent^58^,^59^. In the current study, we presented the novel discovery that CLC-1 degradation is prevented by the Hsp90 inhibitor 17-AAG, which is also being tested in various clinical trials as an anti-cancer agent^39^,^40^,^60^. Together, these data highlight the therapeutic potential of CLC-1 proteostasis modification in treating myotonia patients. In order to further address the clinical significance of our findings, more work is required to verify the specificity, as well as the effectiveness, of MLN4924 and 17-AAG in skeletal muscles. For example, since the biosynthetic mechanism of Na_V1.4_ remains unclear^56^, it will be imperative to determine whether (and perhaps how) these drugs may affect the protein expression and membrane trafficking of the Na^+^ channels in skeletal muscles.

## Methods

### cDNA constructs

Epitope-tagged CLC-1 constructs were generated by subcloning human CLC-1 cDNA into either the pcDNA3 vector (Invitrogen) (for Myc and HA tags)^14^ or the pFlag-CMV2 vector (Sigma) (for Flag tag)^18^. Other cDNA constructs employed in this study include pcDNA3.1-Myc mouse Aha1, pcDNA3-Myc human cullin 4A/4B (Addgene 19951/19922), pcDNA3.1-Myc mouse FKBP8, pcDNA3-HA rat HOP, pcDNA5-V5 human Hsc70 (Addgene 19514), pcDNA5-V5 human Hsp70 (Addgene 19510), pcDNA3-HA human Hsp90α (modified from an original clone kindly provided by Dr. Didier Picard, University of Geneva), and pcDNA3-HA human Hsp90β (Addgene 22847).

### Cell culture and DNA transfection

Human embryonic kidney (HEK) 293T cells were grown in Dulbecco’s modified Eagle’s medium (DMEM) supplemented with 2 mM glutamine, 10% heat-inactivated fetal bovine serum (Hyclone), 100 units/ml penicillin, and 50 μg/ml streptomycin, and were maintained at 37 °C in a humidified incubator with 95% air and 5% CO_2_. Transient transfection was performed by using the Lipofectamine 2000 (LF2000) reagent (Invitrogen). Briefly, cells were plated onto 6- or 12-well plates (for biochemical experiments) or poly-D-lysine-coated coverslips in 24-well plates (for electrophysiological recordings) 24 hrs before transfection. The amount of CLC-1 cDNA used in each well was about 300 (for biotinylation) to 700 (for shRNA knock-down) ng/mL, and the molar ratio for co-transfection (relative to CLC-1 cDNA) ranged from 1 to 3. Various expression constructs were incubated with LF2000 reagent for 20 min at room temperature, and DNA-lipofectamine diluted in Opti-MEM (Invitrogen) was added to culture wells. After 6-hr incubation at 37 °C, the medium was changed and the culture cells were maintained in the 37 °C incubator for 24–48 hrs before being used for biochemical or electrophysiological experiments. Where indicated, drugs [cycloheximide (Sigma) or 17-AAG (Sigma)] were applied to the culture medium.

### RNA interference

Lentivirus-based shRNA constructs (subcloned into the pLKO.1 vector) targeting specific human Aha1 (5′-CCCTGAGAAACATATTGTGAT-3′), FKBP8 (5′-AGTGGACATGACGTTCGAGGA-3′), HOP (5′-CGACCTTCATCAAGGGTTATA-3′), Hsc70 (5′-CGTCTGATTGGACGCAGATTT-3′), or Hsp90β (5′-CTTGTGTTGAAGGCAGTAAAC-3′) sequence were purchased from National RNAi Core Facility, Taiwan. The shRNA for GFP (5′-GACCACCCTGACCTACGGCGT-3′) was used as a control. Recombinant lentivirus was generated by co-transfecting HEK293T cells with the packaging plasmid pCMV-ΔR8.91, the envelope plasmid pMD.G, and shRNA expressing constructs. The virus-containing supernatant was harvested and concentrated by ultracentrifugation to yield the viral stock, which in turn was supplemented with 8 μg/ml of polybrene for infection of HEK293T cells. The infected cells were selected by 5 μg/ml of puromycin and subsequently transfected with the cDNA for CLC-1.

### Immunoblotting

Transfected HEK293T cells were washed twice with ice-cold PBS [(in mM) 137 NaCl, 2.7 KCl, 4.3 Na_2_HPO_4_. 2H_2_O, 1.4 KH_2_PO_4_, pH 7.3] supplemented with 2 mM EDTA, and resuspended in a lysis buffer [(in mM) 150 NaCl, 5 EDTA, 50 Tris-HCl pH7.6, 1% Triton X-100) containing protease inhibitor cocktail. After adding the Laemmli sample buffer to the lysates, samples were sonicated on ice (three times for five seconds each) and heated at 70 °C for 5 min. Samples were then separated by 7.5–10% SDS-PAGE, electrophoretically transferred to nitrocellulose membranes, and detected using rabbit anti-Aha1 (1:2500; Thermo), rabbit anti-CUL4A (1:2000; GeneTex), rabbit anti-CUL4B (1:1000; ProteinTech), rabbit anti-Flag (1:5000; Sigma), rabbit anti-FKBP8 (1:4000; EnoGene), rabbit anti-GAPDH (1:8000; GeneTex), rat anti-HA (1:5000; Roche), rabbit anti-HOP (1:10000; Abcam), rabbit anti-Hsc70 (1:750; Abcam), rabbit anti-Hsp70 (1:10000; GeneTex), rabbit anti-Hsp90β (1:500; Abcam), mouse anti-Myc (clone 9E10), or rabbit anti-α-tubulin (1:5000; GeneTex) antibodies. Blots were then exposed to horseradish peroxidase-conjugated anti-mouse/rabbit IgG (1:5000; Jackson ImmunoResearch) or goat anti-rat IgG (1:5000; Santa Cluz), and revealed by an enhanced chemiluminescence detection system (Thermo Scientific). Results shown are representative of at least three independent experiments. Densitometric scans of immunoblots were quantified by using ImageJ (National Institute of Health).

### Co-immunoprecipitation

Transfected cells were incubated at 37 °C in the presence of 10 μM MG132 for 24 hrs. Cells were solubilized in ice-cold IP buffer [(in mM) 100 NaCl, 4 KCl, 2.5 EDTA, 20 NaHCO_3_, 20 Tris-HCl, pH 7.5, 1 dithiothreitol, 1 PMSF, 1% Triton X-100] containing the protease inhibitor cocktail. Insolubilized materials were removed by centrifugation. Solubilized lysates were pre-cleared with protein G sepharose beads (GE Healthcare Biosciences) for 1 hr at 4 °C, and then incubated for 16 hrs at 4 °C with protein G sepharose beads pre-coated with the anti-Myc or anti-HA antibody. Beads were gently spun down and washed twice in a wash buffer [(in mM) 100 NaCl, 4 KCl, 2.5 EDTA, 20 NaHCO_3_, 20 Tris-HCl, pH 7.5] supplemented with 0.1% Triton X-100, and then twice with the wash buffer. The immune complexes were eluted from the beads by heating at 70 °C for 5 min in the Laemmli sample buffer.

### Biotinylation of cell surface proteins

Transfected cells were washed extensively with D-PBS (Sigma) supplemented with 0.5 mM CaCl_2_, 2 mM MgCl_2_, followed by incubation in 1 mg/ml sulfo-NHS-LC-biotin (Thermo Scientific) in D-PBS at 4 °C for 1 hr with gentle rocking. Biotinylation was terminated by removing the biotin reagents and rinsing three times with 100 mM glycine in PBS, followed by once in TBS buffer [(in mM) 20 Tris-HCl, 150 NaCl, pH 7.4]. Cells were solubilized in a lysis buffer [(in mM) 150 NaCl, 50 Tris-HCl, 1% Triton X-100, 5 EDTA, 1 PMSF, pH 7.6] supplemented with the protease inhibitor cocktail. Insolubilized materials were removed by centrifugation. Solubilized cell lysates were incubated overnight at 4 °C with streptavidin-agarose beads (Thermo Scientific). Beads were washed once in the lysis buffer, followed by twice in a high-salt buffer [(in mM) 500 NaCl, 5 EDTA, 50 Tris-HCl, pH7.6, 0.1% Triton X-100] and once in a low-salt buffer [(in mM) 2 EDTA, 10 Tris-HCl, pH7.6, 0.1% Triton X-100]. The biotin-streptavidin complexes were eluted from the beads by heating at 70 °C for 5 min in the Laemmli sample buffer.

### Protein ubiquitination analyses

Transfected cells were incubated at 37 °C in the presence of 10 μM MG132 for 24 hrs. Cells were solubilized in the IP buffer supplemented with 2.5 mg/ml N-Ethylmaleimide, followed by immunoprecipitation with the anti-Myc antibody as described above.

### Electrophysiological recordings

Conventional whole-cell and cell-attached patch clamp techniques were employed to record CLC-1 Cl^−^ currents. Cells co-transfected with the cDNA for Flag-tagged CLC-1 and pEGFP (molar ratio 1:0.1) were identified with an inverted fluorescence microscope (Leica-DM IRB). Recording electrodes were pulled by a PP-830 puller (Narashige), and displayed a resistance of 2–3 MΩ when filled with the pipette solution. For whole-cell recordings, the pipette solution contained (in mM): 120 CsCl, 10 EGTA, 10 HEPES, pH 7.4; while the bath solution comprised (in mM): 140 NaCl, 4 CsCl, 2 MgCl_2_, 2 CaCl_2_, 10 HEPES, pH 7.4. Where indicated, 5 mM Mg-ATP was freshly prepared on the day of experiment and added to the pipette solution. For cell-attached recordings, the pipette solution was the same as the whole-cell bath solution. The bath solution contained (in mM): 130 KCl, 5 MgCl_2_, 1 EGTA, 10 HEPES, pH 7.4. Data were acquired and digitized with Axopatch 200B and Digidata 1322A, respectively, via pCLAMP 9.0 (Molecular Devices). Cell capacitances were measured using the built-in functions of pCLAMP 9.0 and were compensated electronically with Axopatch 200B. The holding potential was set at 0 mV. Data were sampled at 2 kHz and filtered at 1 kHz. All recordings were performed at room temperature (20–22 °C).

### Statistical analyses

All values were presented as mean ± SEM. The significance of the difference between two means was tested using the Student’s *t* test, whereas means from multiple groups were compared using the one-way ANOVA analysis. All statistical analyses were performed with Origin 7.0 (Microcal Software).

## Additional Information

**How to cite this article**: Peng, Y.-J. *et al*. Regulation of CLC-1 chloride channel biosynthesis by FKBP8 and Hsp90β. *Sci. Rep.* **6**, 32444; doi: 10.1038/srep32444 (2016).

## Supplementary Material

Supplementary Information
