# Supplementary material for: Regulation of CLC-1 chloride channel biosynthesis by FKBP8 and Hsp90β
Source: Sci Rep. 2016 Sep 1;6:32444. doi: 10.1038/srep32444 (PMC5007535; doi:10.1038/srep32444)

**Supplementary Information**

**Regulation of CLC-1 chloride channel biosynthesis by FKBP8 and Hsp90β**

Yi-Jheng Peng, Jing-Jia Huang, Hao-Han Wu, Hsin-Ying Hsieh, Chia-Ying Wu, Shu-Ching Chen, Tsung-Yu Chen, and Chih-Yung Tang

**SUPPLEMENTARY METHODS**

***Yeast two-hybrid screening***

The DupLEX-A yeast two-hybrid system (OriGene) was used to screen a mouse skeletal muscle cDNA library (OriGene). The carboxyl-terminal region of human CLC-1 (amino acids 593-871, also known as the C2 fragment) was used as the bait to screen the library. The cDNA for CLC-1 C2 fragment was amplified by PCR and fused in-frame to the coding sequence for the DNA-binding protein LexA that was subcloned into the yeast expression plasmid pGilda. The yeast strain EGY48, which contains the reporter gene LEU2 downstream of the LexA-operator, was sequentially transformed (using the lithium acetate method) with *i*) the bait-containing plasmid pGilda, *ii*) the reporter plasmid pSH18-34 (containing the LexA operator-lacZ fusion gene), and *iii*) an activation domain-fused cDNA library subcloned in the plasmid pJG4-5. After incubating at 30˚C for 2-7 days, transformed yeast colonies growing on leucine dropout plates were scored positive for interacting proteins. Positive colonies were further selected by the β-galactosidase assay. Plasmid DNA was extracted from yeast colonies and used to transform the *E. coli* strain DH5α. Candidate cDNA clones were screened by PCR with pJG4-5-specific primers, followed by online (BLAST) and in-house sequence analyses.

In addition, the carboxyl-terminal region of mouse CLC-2 (amino acids 581-794) was also employed for yeast two-hybrid screening of a rat brain cDNA library (OriGene) by following the identical procedure as described above.

***Glutathione S-transferase (GST) pull-down assays***

GST fusion proteins were produced and purified by following the manufacturer’s instruction (Stratagene). In brief, cDNA fragments encoding three different carboxyl-terminal regions of the human CLC-1 [C1, amino acids 593-988; C2, amino acids 593-871; C3, amino acids 820-988] were subcloned into the pGEX vector (GE Healthcare) and expressed in the *E. coli* strain BL21*.* Bacterial cultures were grown at 30°C, induced with 0.1 mM isopropyl-β-D-thiogalactopyranoside, and then harvested by centrifugation at 8,000x*g* for 10 min at 4ºC. Cell pellets were resuspended in the B-PER reagent (Pierce) containing 1 mM phenylmethylsulfonyl fluoride (PMSF) and protease inhibitor cocktail (Roche Applied Science). The lysates were clarified by centrifugation at 15,000x*g* for 15 min, and glutathione-agarose beads (Sigma) were used to bind the GST fusion proteins from the supernatant. GST protein-coated beads (4–8 μg) were incubated with pre-cleared *in vitro* translated proteins or cell lysates at 4ºC overnight. The bead-protein complexes were then washed with buffer A [(in mM) 100 NaCl, 4 KCl, 2.5 EDTA, 20 NaHCO_3_, 20 Tris-HCl, pH 7.5, plus 1 PMSF, 1 Na_3_VO_4_, 1 NaF, 1 β-glycerophosphate] (with and without 1% Triton X-100), and the proteins were eluted by boiling for 5 min in the Laemmli sample buffer. *In vitro* protein translation was performed by using the TNT transcription-translation system (Promega).

***Analyses of steady-state voltage-dependent property of CLC-1 channels***

Patch clamp recordings in HEK293T cells were conducted to obtain the steady-state voltage-dependence of the open probability (*P*_o_–V curve) of the fast and the common gates of CLC-1 channels. The holding potential was 0 mV. To estimate the *P*_o_ of both fast and common gates (Overall *P*_o_; *P_f_ x P_c_*), the standard voltage protocol (see Fig. 7A) comprised test pulses ranging from +100 mV to -140 mV in -20 mV steps, followed by a second voltage step (tail potential) to -100 mV for 200 ms. The values of the initial tail current amplitudes at -100 mV following various test pulses, determined by fitting tail currents to a double-exponential function, were then normalized to the maximal initial tail current amplitude obtained from the +100-mV test pulse. For the *P*_o_ of common gates (Common *P*_o_; *P_c_*), the -100-mV tail potential was preceded by a 1-ms +170-mV pulse to fully activate fast gates. The values of the initial tail current amplitudes were also normalized to the maximal initial tail current amplitude induced by the +100-mV test pulse. The solid lines shown in Figure 7 and Supplementary Figure S3 represent curve fitting of *P*_o_-V curves with a Boltzmann equation: *P*_o_ = *P_min_* + (1-*P_min_*) / {1+exp[(V_0.5_-V)/*k*]}, where V_0.5_ and *k* is the half-activating voltage and slope factor, respectively, for the corresponding *P*_o_-V curve.

**SUPPLEMENTARY REFERENCES**

[1] B. Bennetts, G.Y. Rychkov, H.L. Ng, C.J. Morton, D. Stapleton, M.W. Parker, B.A. Cromer, Cytoplasmic ATP-sensing domains regulate gating of skeletal muscle ClC-1 chloride channels, J Biol Chem, 280 (2005) 32452-32458.

[2] B. Bennetts, M.W. Parker, B.A. Cromer, Inhibition of skeletal muscle ClC-1 chloride channels by low intracellular pH and ATP, J Biol Chem, 282 (2007) 32780-32791.

[3] T.T. Lee, X.D. Zhang, C.C. Chuang, J.J. Chen, Y.A. Chen, S.C. Chen, T.Y. Chen, C.Y. Tang, Myotonia congenita mutation enhances the degradation of human CLC-1 chloride channels, PLoS One, 8 (2013) e55930.

***Supplementary Table S1. Quantification of the effect of FKBP8 or Aha1 co-expression on CLC-1 WT and A531V mutant.*** (related to Figure 1)

Data were collected from 3-5 independent experiments and are displayed as mean ± SEM. Numbers in parentheses denote the number of observation.

| Co-expression conditions | Mean relative protein levels (Fig. 1A) | |
| --- | --- | --- |
|  | WT | A531V |
| FKBP8 | 2.5 ± 0.6 (12) | 3.7 ± 0.6(8) |
| Aha1 | 2.5 ± 0.3 (12) | 2.4 ± 0.4 (10) |

| Co-expression conditions | Mean relative surface expression levels (Fig. 1B) | |
| --- | --- | --- |
|  | WT | A531V |
| FKBP8 | 3.8 ± 0.5 (4) | 9.9 ± 0.6 (3) |
| Aha1 | 1.7 ± 0.2(6) | 1.5 ± 0.2 (8) |

| Co-expression conditions | Mean relative membrane trafficking ratios (Fig. 1B) | |
| --- | --- | --- |
|  | WT | A531V |
| FKBP8 | 1.8 ± 0.2 (4) | 2.4 ± 0.2 (3) |
| Aha1 | 1.1 ± 0.2 (6) | 0.8 ± 0.1 (8) |

| shRNA knock-down | Mean relative protein levels (Fig. 1C) | |
| --- | --- | --- |
|  | WT | A531V |
| FKBP8 | 1.1 ± 0.1 (5) | 1.1 ± 0.04 (5) |
| Aha1 | 0.8 ± 0.04 (16) | 0.7 ± 0.1 (12) |

***Supplementary Table S2. Quantification of CLC-1 protein half-life.*** (related to Figures 4 and 5)

Protein half-life values of CLC-1 WT and A531V mutant under various experimental conditions were determined as follows. Mean protein densities in response to different CHX treatment durations were standardized as the ratio of CLC-1 signals to the cognate tubulin signals, followed by normalization to those of no-treatment controls at 0 hr. Mean protein half-life values were then estimated from linear-regression analyses of the semi-logarithmic plot of the protein degradation time course. Data were collected from 3-6 independent experiments. Numbers in parentheses denote the number of observation.

| Co-expression or drug treatment conditions | Protein half-life (hours) | |
| --- | --- | --- |
|  | WT | A531V |
| Vector | 6.6 (4) | 3.6 (8) |
| FKBP8 | 10.1 (4) | 8.0 (9) |
| Aha1 | 8.8 (2) | 4.1 (10) |
| HOP | − | 3.3 (5) |
| Hsc70 | − | 3.9 (7) |
| Hsp90β | − | 4.0 (8) |
| DMSO | 7.4 (11) | 3.9 (5) |
| 17-AAG | 8.1 (11) | 8.5 (3) |

***Supplementary Table S3. Quantification of the effect of 17-AAG treatment on protein levels.*** (related to Figure 5)

CLC-1 protein levels in response to different concentrations of 17-AAG treatment were normalized with respect to the corresponding DMSO control. Data were collected from 3-7 independent experiments and are displayed as mean ± SEM. Numbers in parentheses denote the number of observation.

|  | Mean relative protein levels in response to different 17-AAG concentrations | | | | | | |
| --- | --- | --- | --- | --- | --- | --- | --- |
|  | 0.1 μM | 0.2 μM | 0.5 μM | 0.8 μM | 1.0 μM | 2.5 μM | 5 μM |
| CLC-1 WT  (5-14) | 1.1 ± 0.1 | 1.1 ± 0.1 | 1.6 ± 0.1 | 1.7 ± 0.2 | 2.3 ± 0.2 | 2.7 ± 0.3 | 4.6 ± 0.5 |
| CLC-1 A531V  (5-12) | 1.0 ± 0.1 | 0.9 ± 0.1 | 1.3 ± 0.1 | 1.5 ± 0.1 | 2.3 ± 0.2 | 2.6 ± 0.5 | 3.6 ± 0.5 |
| CUL4A  (4-13) | 1.0 ± 0.1 | 1.1 ± 0.1 | 1.0 ± 0.1 | 0.9 ± 0.2 | 0.8 ± 0.1 | 0.5 ± 0.03 | 0.5 ± 0.1 |
| CUL4B  (4-14) | 1.0 ± 0.1 | 1.0 ± 0.1 | 0.7 ± 0.1 | 0.6 ± 0.1 | 0.5 ± 0.1 | 0.3 ± 0.1 | 0.3 ± 0.1 |
| FKBP8  (2-4) | − | − | 1.4 ± 0.1 | − | 1.1 ± 0.2 | 1.1 ± 0.1 | 0.9 ± 0.03 |
| Aha1  (6-11) | − | − | 1.3 ± 0.2 | − | 1.2 ± 0.1 | 1.2 ± 0.1 | 1.3 ± 0.1 |
| HOP  (2-3) | − | − | 1.2 ± 0.1 | − | 1.1 ± 0.1 | 1.0 ± 0.1 | 1.1 ± 0.1 |
| Hsc70  (2-4) | − | − | 1.3 ± 0.3 | − | 1.1 ± 0.03 | 1.1 ± 0.1 | 0.9 ± 0.1 |
| Hsp70  (2-8) | − | − | 2.0 ± 0.02 | − | 2.4 ± 0.4 | 2.2 ± 0.5 | 2.8 ± 0.6 |
| Hsp90α  (2) | − | − | 1.0 ± 0.2 | − | 0.8 ± 0.1 | 1.1 ± 0.2 | 0.9 ± 0.1 |
| Hsp90β  (2) | − | − | 0.9 ± 0.1 | − | 0.9 ± 0.1 | 0.9 ± 0.2 | 1.0 ± 0.3 |

***Supplementary Figure S1. Physical association of CLC-1 with FKBP8 and Aha1.*** (related to Figure 1)

Biochemical demonstration of the interaction of CLC-1 with FKBP8 and Aha1 in HEK293T cells. ***(A)*** GST pull-down of Myc-tagged FKBP8 (Myc-FKBP8) (*left*) or Aha1 (Myc-Aha1) (*right*) with the indicated GST-CLC-1 carboxyl-terminal fusion proteins (C1, C2, C3). Lysates from HEK293T cells over-expressing Myc-tagged constructs were employed for the pull-down assay, followed by immunoblotting with the anti-Myc antibody (α-Myc). The molecular weight markers (in kiloDaltons) are labeled to the left. ***(B)*** Co-immunoprecipitation of Myc-FKBP8 (*left*) or Myc-Aha1 (*right*) with Flag-tagged CLC-1 (Flag-CLC-1). Co-expression with the Myc vector was used as the control experiment. Cell lysates were immunoprecipitated (IP) with α-Myc, followed by immunoblotting (IB) with the indicated antibodies (α-Myc or α-Flag). Corresponding expression levels of FKBP8, Aha1, and CLC-1 in the lysates are shown in the *Input* lane. Input represents about 10% of the total protein used for immunoprecipitation.


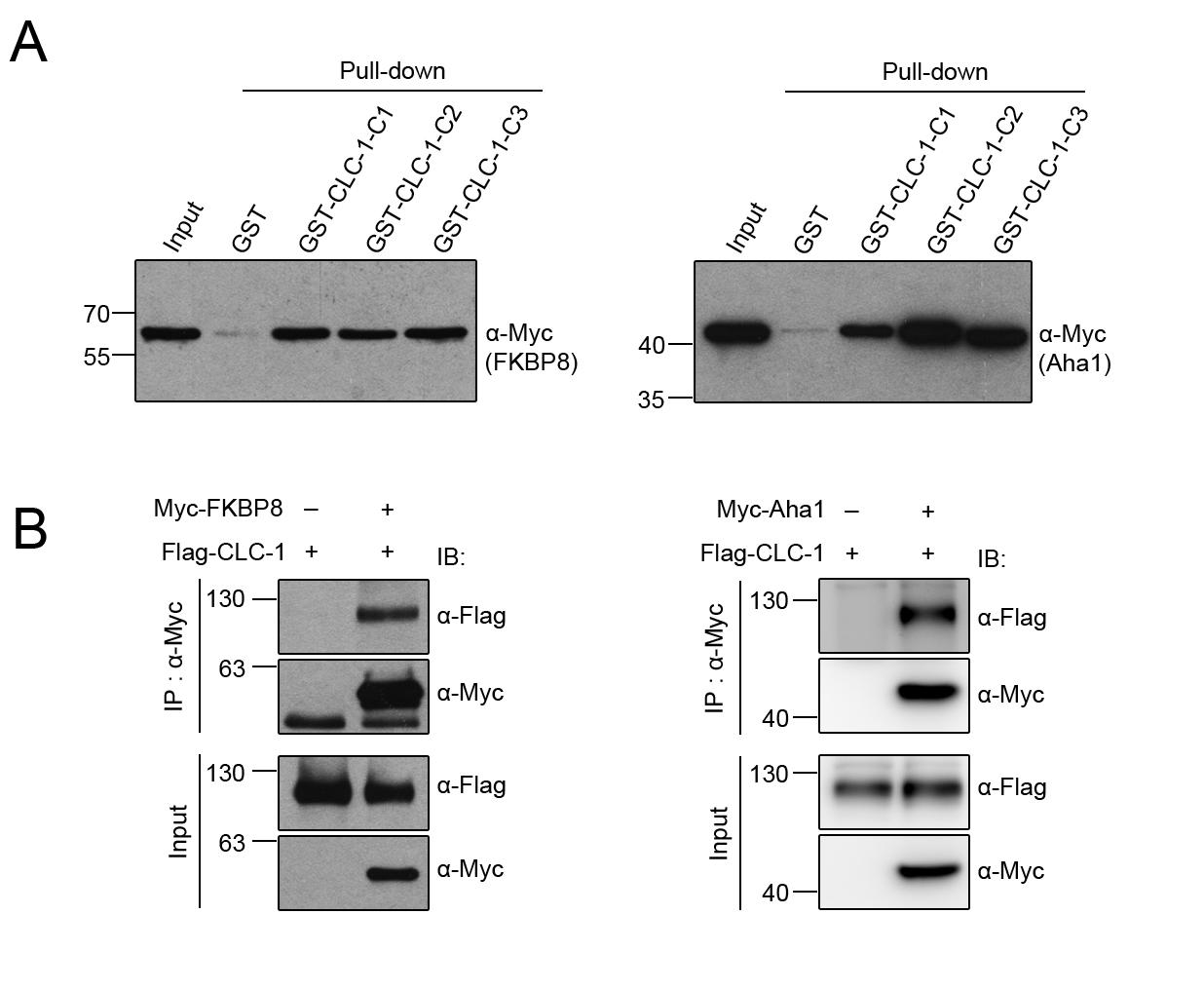


***Supplementary Figure S2. Physical association of CLC-1 with HOP, Hsc70, and Hsp90β.*** (related to Figures 2 and 3)

Biochemical demonstration of the interaction of CLC-1 with HOP, Hsc70, and Hsp90β in HEK293T cells. ***(A)*** (*Left*) GST pull-down of HA-tagged HOP (HA-HOP) with GST-CLC-1 fusion proteins. (*Right*) Co-immunoprecipitation of HA-HOP with Flag-CLC-1. ***(B)*** (*Left*) GST pull-down of V5-tagged Hsc70 (V5-Hsc70) with GST-CLC-1 fusion proteins. (*Right*) Co-immunoprecipitation of V5-Hsc70 with Flag-CLC-1. ***(C)*** GST pull-down of HA-tagged Hsp90β (HA-Hsp90β) with GST-CLC-1 fusion proteins. *Input* represents about 10% of the total protein used for immunoprecipitation.


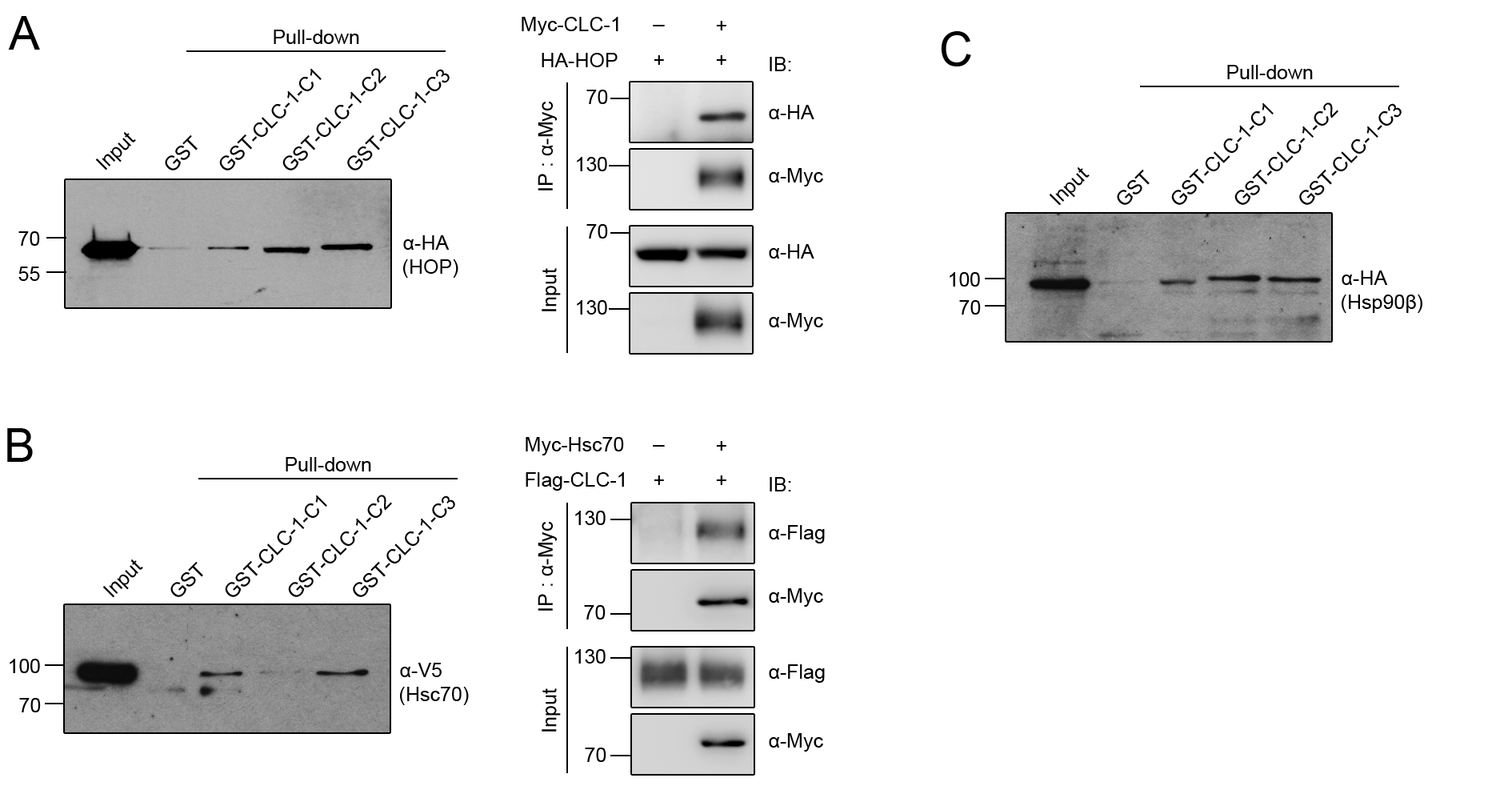


***Supplementary Figure S3. Modulation of the voltage-dependence property of CLC-1 A531V by intracellular ATP.*** (related to Figure 7)

Whole-cell patch clamp analyses of the effect of intracellular ATP on CLC-1 A531V mutant channels in HEK293T cells. “Control” refers to the application of standard ATP-free pipette solution, which can diffuse into HEK293T cells (also known as the wash-in process) and, following the establishment of diffusion equilibrium, becomes the effective intracellular solution. “5 mM ATP” denotes the presence of additional ATP in the pipette solution. The effect of intracellular ATP was analyzed for the *P*_o_ of fast and common gates (Overall *P*_o_) and the *P*_o_ of common gates (Common *P*_o_). Our results support the notion that high concentration of intracellular ATP markedly shifts the voltage gating of CLC-1 A531V to more positive potentials. Identical observations were also reported for CLC-1 WT (Supplementary References [1,2]). Under the whole-cell recording mode, CLC-1 WT and A531V mutant display comparable *P*_o_-V curves in HEK293T cells (see Fig. 2E in Supplementary Reference [3]). In the current study, CLC-1 WT was recorded in the cell-attached configuration, which means that the cytoplasmic side of these channels was exposed to the physiological ATP concentration in HEK293T cells. By contrast, the A531V mutant was recorded in the whole-cell configuration, which contains significantly less ATP due to the wash-in of the ATP-free pipette solution. Therefore, the apparently inconsistent half-activating voltages (V_0.5_) between WT and A531V, as implied by the *P*_o_-V curves shown in Figure 7, most likely reflect the different cytoplasmic ATP concentrations between the two patch clamp recording modes, rather than the presence of a divergent voltage-dependence property between the WT and the mutant channels.


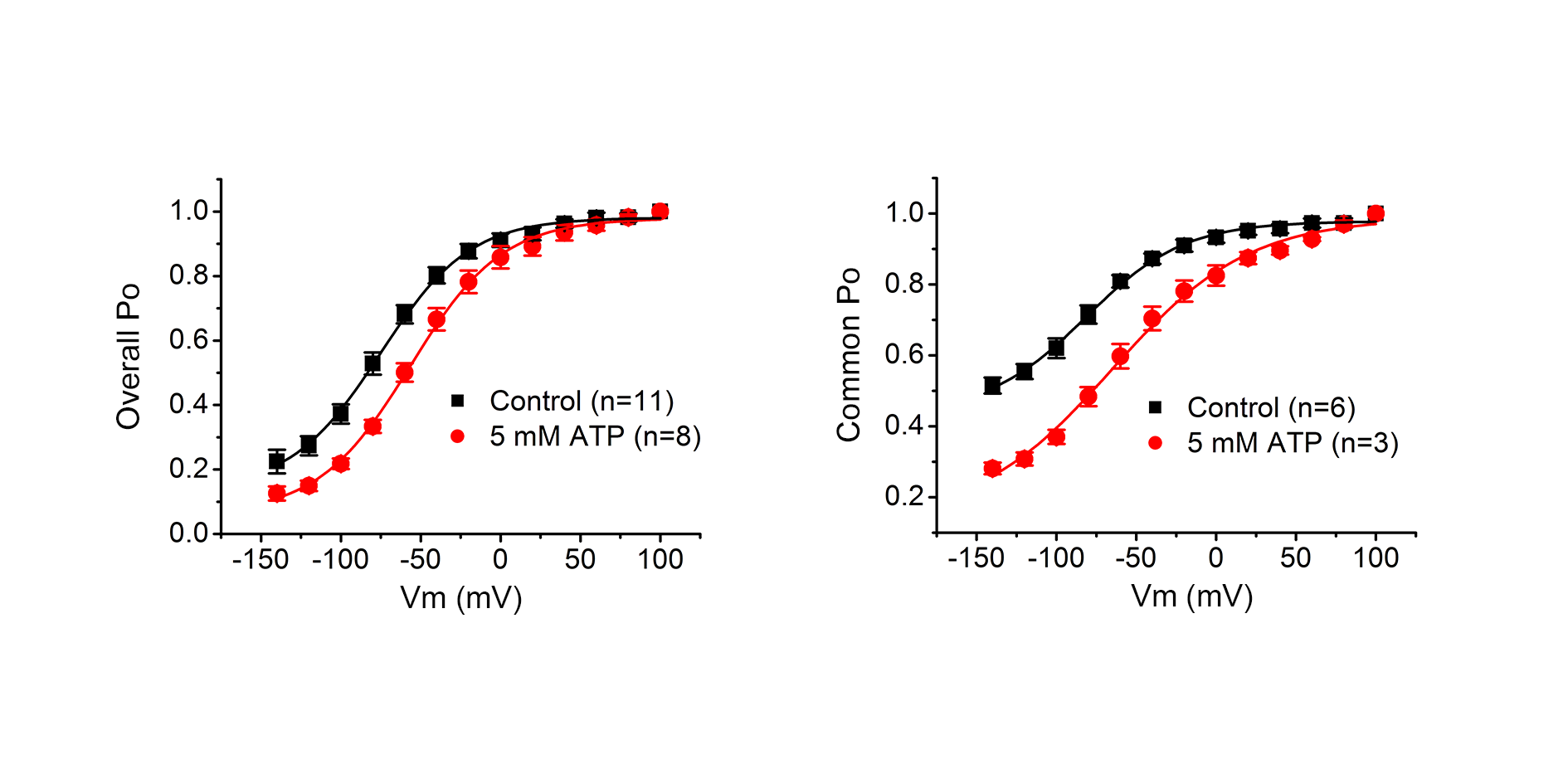


***Supplementary Figure S4. Uncropped images of the immunoblots presented in the main figures.***

(related to Figure 1)


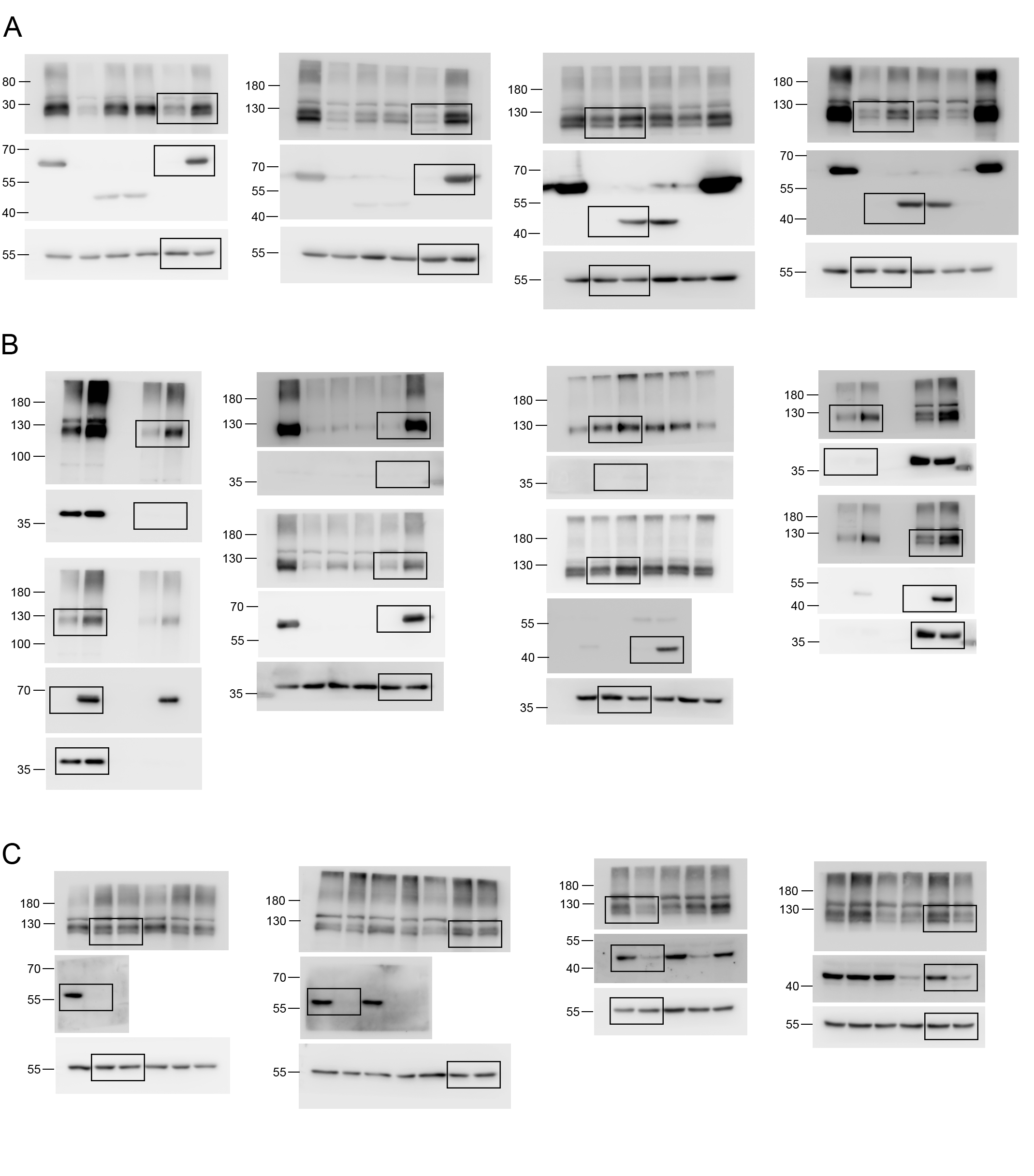


(related to Figure 2)


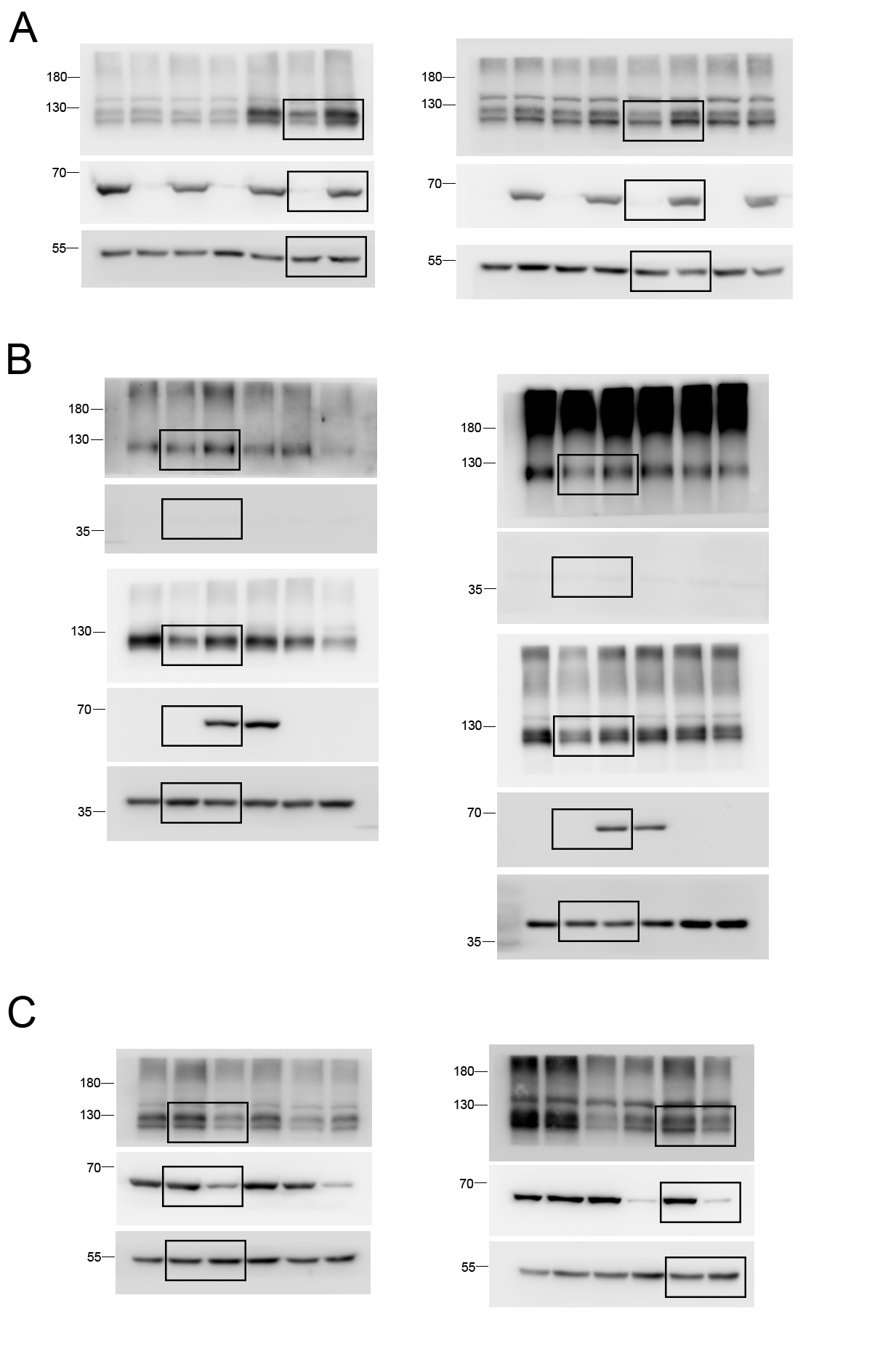


(related to Figure 3)


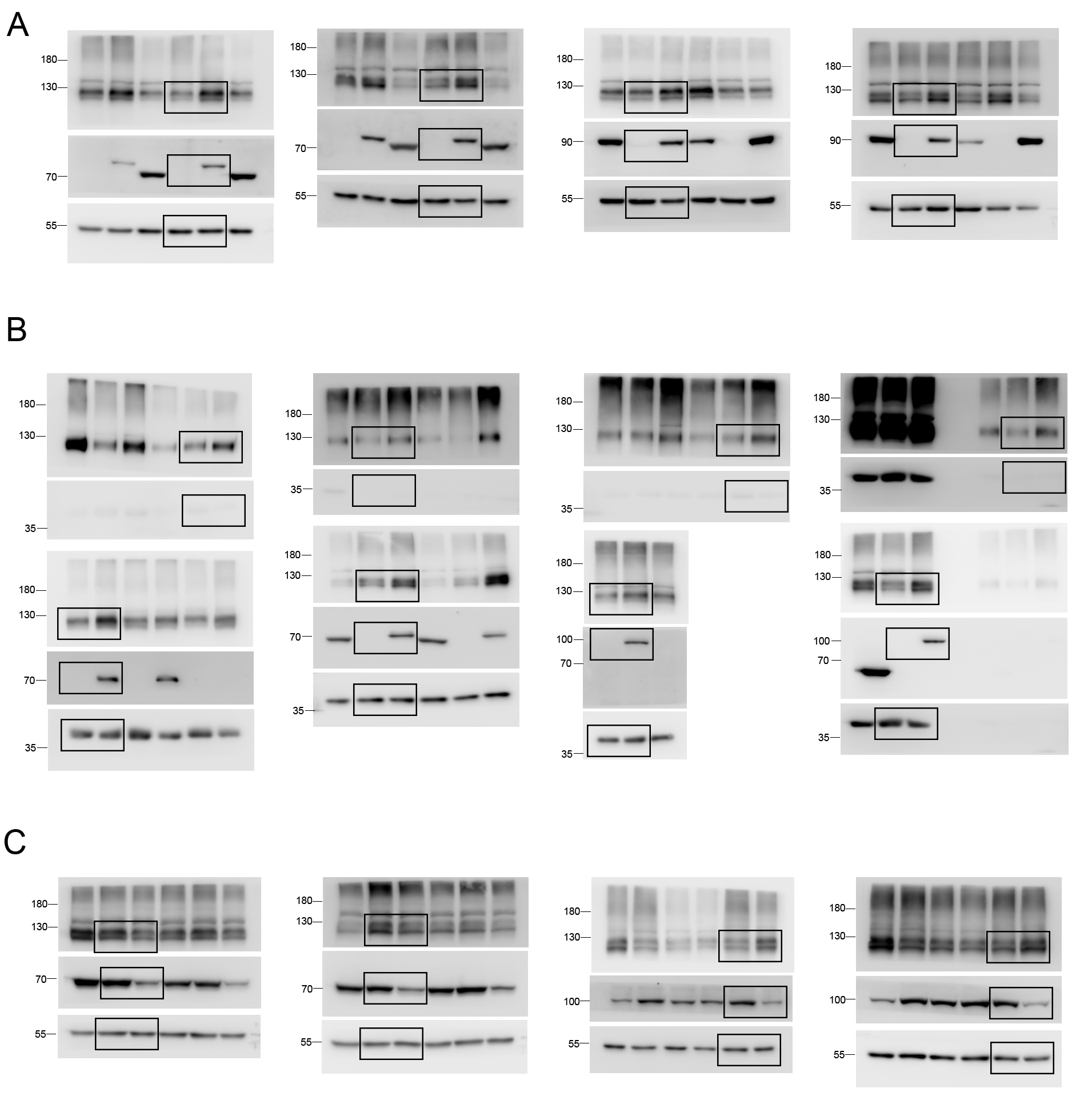


(related to Figure 4)


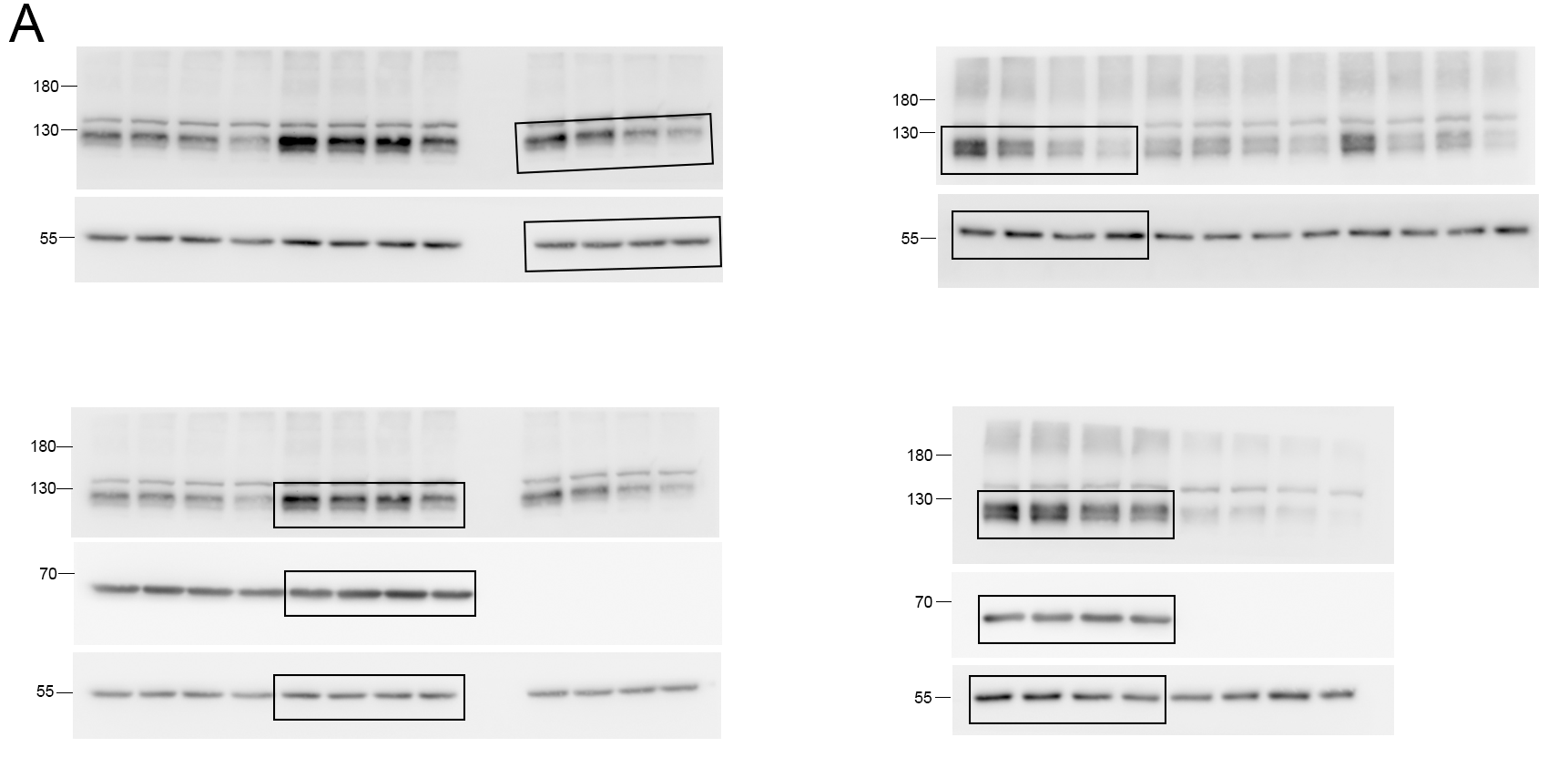


(related to Figure 5)


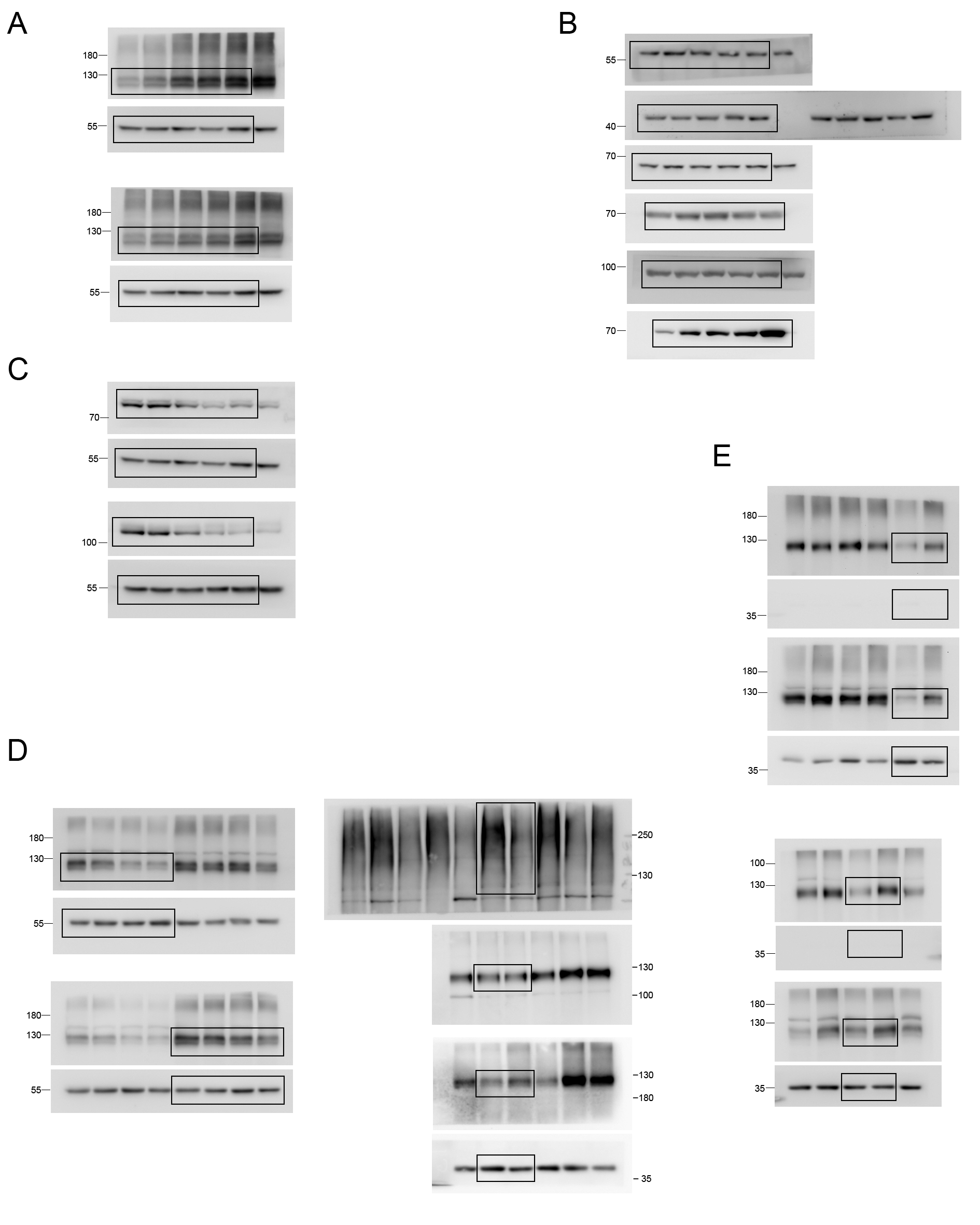


(related to Figure 6)


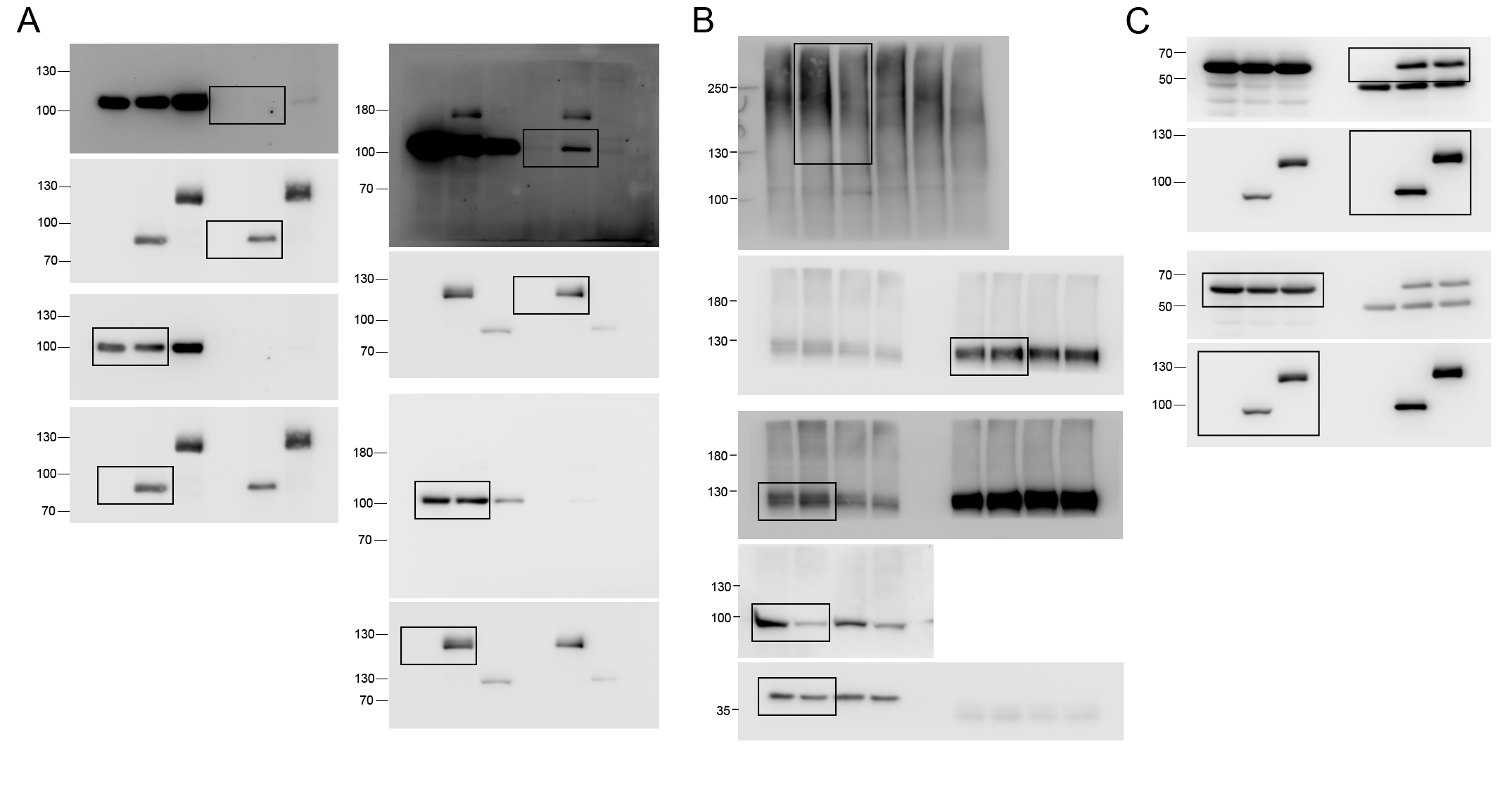

Supplement: Supplementary Information [file srep32444-s1.doc]
